# Common Promoter Elements in Odorant and Vomeronasal Receptor Genes

**DOI:** 10.1371/journal.pone.0029065

**Published:** 2011-12-28

**Authors:** Jussara S. Michaloski, Pedro A. F. Galante, Maíra H. Nagai, Lucia Armelin-Correa, Ming-Shan Chien, Hiroaki Matsunami, Bettina Malnic

**Affiliations:** 1 Departamento de Bioquímica, Instituto de Química, Universidade de São Paulo, São Paulo, São Paulo, Brazil; 2 Ludwig Institute for Cancer Research, São Paulo, São Paulo, Brazil; 3 Department of Molecular Genetics and Microbiology and Neurobiology, Duke University Medical Center, Durham, North Carolina, United States of America; German Institute for Human Nutrition, Germany

## Abstract

In mammals, odorants and pheromones are detected by hundreds of odorant receptors (ORs) and vomeronasal receptors (V1Rs and V2Rs) expressed by sensory neurons that are respectively located in the main olfactory epithelium and in the vomeronasal organ. Even though these two olfactory systems are functionally and anatomically separate, their sensory neurons show a common mechanism of receptor gene regulation: each neuron expresses a single receptor gene from a single allele. The mechanisms underlying OR and VR gene expression remain unclear. Here we investigated if OR and V1R genes share common sequences in their promoter regions.

We conducted a comparative analysis of promoter regions of 39 mouse V1R genes and found motifs that are common to a large number of promoters. We then searched mouse OR promoter regions for motifs that resemble the ones found in the V1R promoters. We identified motifs that are present in both the V1R and OR promoter regions. Some of these motifs correspond to the known O/E like binding sites while others resemble binding sites for transcriptional repressors. We show that one of these motifs specifically interacts with proteins extracted from both nuclei from olfactory and vomeronasal neurons. Our study is the first to identify motifs that resemble binding sites for repressors in the promoters of OR and V1R genes. Analysis of these motifs and of the proteins that bind to these motifs should reveal important aspects of the mechanisms of OR/V1R gene regulation.

## Introduction

In mammals, olfactory stimuli are basically detected by sensory neurons located in two different organs: the main olfactory system and the vomeronasal organ (VNO) [1], [2], [3], [4]. Volatile odorants are detected by odorant receptors (ORs) expressed in the olfactory sensory neurons located in the olfactory epithelium [5]. The VNO specifically detects pheromones, chemical signals that elicit a series of innate social behaviors, such as mating and aggression. Pheromones are sensed by two distinct families of vomeronasal receptors, the V1Rs and V2Rs, which are respectively expressed in sensory neurons located in the apical and basal layers of the vomeronasal epithelium [6], [7], [8], [9]. Pheromones can also be detected by small families of chemosensory receptors, the trace amine-associated receptors (TAARs) [10], which are expressed in the olfactory epithelium, and the formyl peptide receptors (FPRs) [11], [12], which are expressed in the VNO.

The mouse V1R family consists of about 187 intact genes, which can be subdivided into 12 divergent subfamilies [13], [14], [15], [16]. Even though V1Rs show no significant sequence identities with ORs, the pattern of V1R expression shares striking similarities with the expression of ORs. Each olfactory sensory neuron expresses one single odorant receptor (OR) gene out of ∼1000 genes [17], [18], [19], [20], [21]. Analogously, individual vomeronasal neurons express one single V1R gene [6], [22], [23]. Olfactory neurons that express an OR gene that does not have an intact open reading frame, and therefore cannot be translated into a functional OR protein, can express a second OR gene [24], [25], [26]. These results indicate that a post-translational feedback is required to prevent the expression of other OR genes. Similarly, the expression of a non-functional V1R gene leads to the expression of other functional V1R genes [23], [27]. Also, the transcription of an exogenous OR coding sequence from a V1R promoter in vomeronasal neurons is able to prevent the transcription of all endogenous V1R genes [27], indicating that a common negative feedback mechanism is used by the two chemosensory systems.

In studies using transgenic mice, it was demonstrated that minimal proximal promoter regions are sufficient to drive OR gene expression similar to that of the endogenous gene [28], [29], [30], [31]. If the same case is true for V1R gene promoters, has not been determined yet. Analysis of the promoter regions of several OR genes revealed that the vast majority of these promoters have O/E like and homeodomain binding sites [30], [32], [33], [34], [35], [36]. Mutation of these sites in the endogenous M71 OR locus does not abolish M71 gene expression, but results in a reduced number of M71-expressing neurons and a more ventralized epithelial pattern [29]. Altogether, these results indicate that motifs in proximal promoter regions are involved in OR gene regulation.

Very little is known about the V1R gene promoter regions. Regions of homology were identified in the promoter regions of 15 V1R genes located in a cluster on the mouse chromosome 6D locus [37]. These conserved promoter sequences, however, are specific to the V1Rs at the 6D locus, they were not found in the promoter regions of V1R genes located in the other locus in chromosome 6 [37]. It was demonstrated that members within each one of the V1R subfamilies share a broad and conserved promoter region, while members belonging to different subfamilies show no evident homology in their promoter regions [38].

Here we have compared the promoter regions of V1R and OR genes. First we have determined the promoter sequences for 39 V1R genes by using RLM-RACE. Then, we searched these promoters for common elements. We found motifs that are also present in the promoters of OR genes. Some of these motifs correspond to the known O/E like binding sites and others resemble the binding sites for transcription factors that were shown to work as repressors and belong to the BTB-ZF family. We show that one of the motifs is able to form a complex with nuclear proteins extracted from the VNO and OE, but not from other unrelated tissues. We also show that Zbtb7b is the member of the BTB-ZF family that is predominantly expressed in the olfactory epithelium.

## Results

### Generation of V1R cDNAs

**Figure 1:**
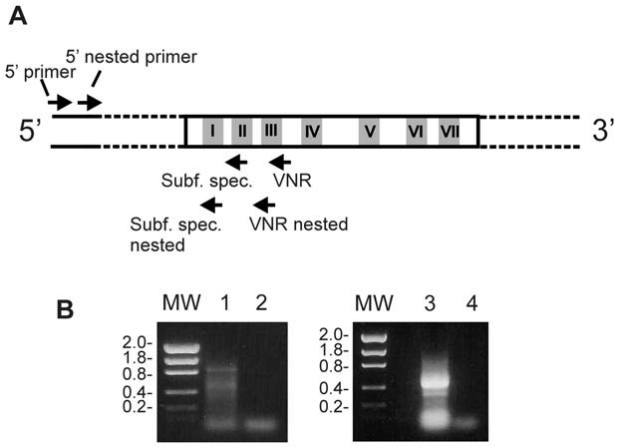
Generation of 5′ cDNAs for V1Rs. A) Schematic representation of a V1R cDNA showing the seven transmembrane regions (I–VII) and the regions matched by the degenerate primers (VNR and VNR nested), the subfamily specific primers and the GeneRacer primers. Complete 5′ end cDNAs corresponding to V1R sequences were amplified by PCR using the following combinations of primers: 5′ primer plus the degenerate VNR primer followed by a nested PCR using 5′ nested primer plus the degenerate VNR nested primer; or 5′ primer plus a subfamily specific primer followed by a nested PCR using 5′ nested primer plus a subfamily specific nested primer. B) The PCR products obtained by using 5′primer and VNR (lane 1) and nested PCR using 5′ nested primer and VNR nested (lane 3) are shown. The corresponding negative controls (no cDNA added) are shown in lanes 2 and 4. Molecular weights (MW) are given in kilobases.

We performed RNA ligase-mediated rapid amplification of 5′ cDNA ends (RLM-RACE) using total RNA purified from mouse vomeronasal epithelium to obtain complete 5′-end sequences from V1R cDNAs. This method has the advantage that only full-length transcripts are amplified [36]. To specifically amplify V1R cDNAs we used both degenerate primers matching to conserved regions in V1Rs, and also specific primers, matching to conserved regions within the different subfamilies (Fig. 1). Because V1R sequences are highly divergent between subfamilies [13], and consequently there are very few conserved sequence motifs which are present in nearly all family members, we could not amplify a large number of different V1Rs by using the degenerate primers, but by using the subfamily specific primers we were able to amplify members of all the V1R subfamilies, except for subfamily l, which contains one single member [13] (Table 1).

**Table 1:** Subfamily distribution of V1R genes analyzed in this study.

V1R subfamily	Annotated V1R genes	V1R genes (cDNAs)
V1Ra	10	2
V1Rb	9	5
V1Rc	31	14
V1Rd	22	1
V1Re	13	1
V1Rf	5	2
V1Rg	12	4
V1Rh	21	5
V1Ri	10	3
V1Rj	2	1
V1Rk	1	1
V1Rl	1	0
Total	137	39

The number of annotated mouse V1R genes from [13] and of mouse V1R genes for which cDNA sequences were obtained in the present study are shown for each one of the V1R subfamilies.

### V1R gene structures

428 cDNA clones were sequenced (GenBank:EX601137–EX601479) and we used BLAT [39] and Sim4 [40] to align these cDNA sequences with the mouse genome sequence (mm9, retrieved from UCSC Genome Browser (http://genome.ucsc.edu)). The previously annotated mouse V1R sequences [13], RefSeq (http://www.ncbi.nlm.nih.gov/RefSeq/) were also included in the alignment, to help with the localization of the cDNA's 5′ regions. Each cDNA sequence aligned to one single genomic region, and the cDNAs sharing genomic coordinates with one of the annotated V1R genes were considered to correspond to that particular V1R gene. In this way, we obtained 39 V1R clusters, where each cluster corresponds to one different V1R gene. Each cluster contains at least 4 cDNA sequences.

The structural organization of the 39 V1R genes is shown in Figure S1. Most of the V1R genes (37 out of 39) have at least one 5′-UTR exon, which range from 22 to 518 bp in size and are on average 146 bp long. Accordingly, most of the V1R genes have at least one 5′ intron, with an average size of 5 kb and ranging from 237 bp to 39 kb in size.

### Analysis of V1R gene promoter regions

**Figure 2:**
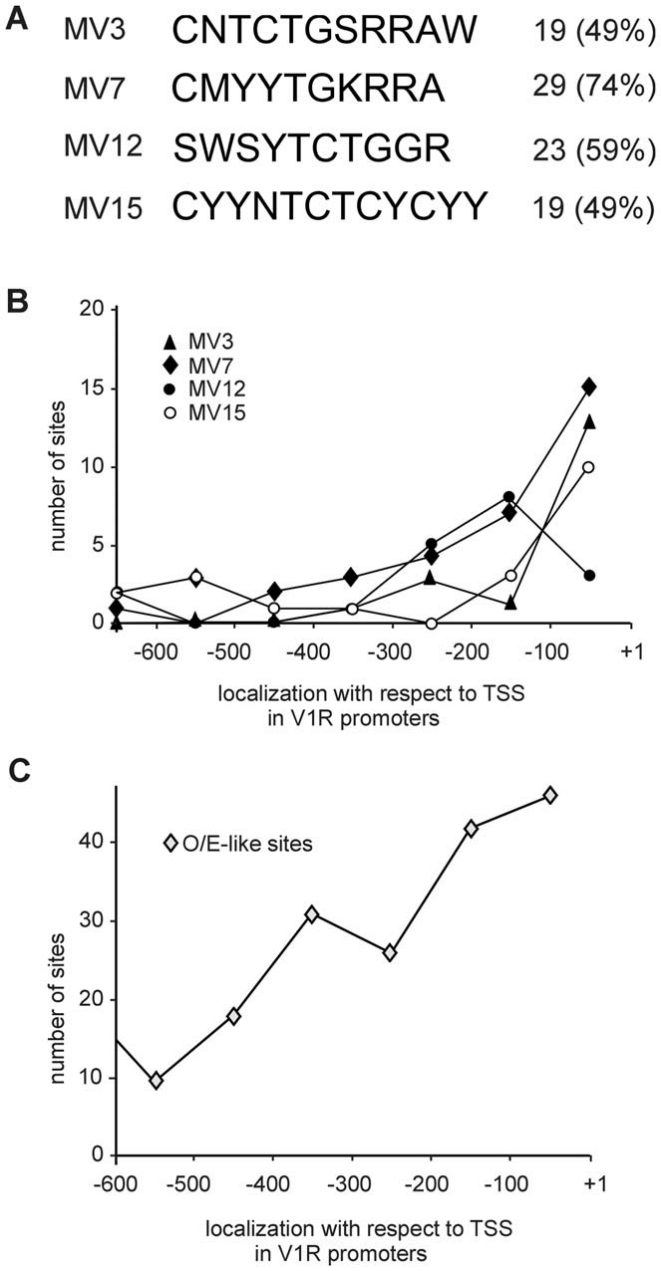
Motifs found in V1R promoter regions. A) Motif consensus sequences identified by using Gibbs sampler are shown. The sequences follow the IUPAC degeneracy code: R (A or G), S (C or G), W (A or T), Y (C or T) and K (G or T). The number (%) of V1R gene promoters containing each one of the motifs is shown. B) The total number of sites for the different identified motifs found across the V1R promoter regions is shown. The positions of the TSS (+1) and 600 bp upstream of the TSS (−100 to −600) are indicated. C) The total number of O/E-like sites (as described in Michaloski et al., 2006) found across the V1R promoter regions is also shown.

The transcription start sites (TSSs) for the 39 V1R genes were determined and sequences (1 kb long) located upstream of each TSS were excised from the mouse genome sequence and screened for motifs that are common to a large fraction of the promoter sequences. To do this, we used the Gibbs recursive sampler and the Consensus tools, which were designed to identify common elements in collections of unaligned DNA sequences. We found motifs that are shared by a large fraction of the V1R promoter sequences (Fig. 2A). These motifs were also selected based on their spatial distribution in the promoter regions: they are all concentrated near the TSSs (between +1 and −300 bp) of the respective V1R genes (Fig. 2B).

We have previously shown that the majority of the OR promoter regions have binding sites for the Olf-1 like proteins (O/E proteins) [36]. Because it is known that the *Olf-1* (*O/E-1*), *olf-2* (*O/E-2*) and *olf-3* (*O/E-3*) (but not *olf-4* (*O/E-4*)) genes are expressed in the VNO [41], [42], we also analyzed whether V1R promoter regions contain the O/E like binding sites M1–M4 described in [36]. We found that all the 39 V1R genes have O/E like sites, and that the sites are also concentrated near the TSSs (Fig. 2C). The new motifs shown in Figure 2A do not overlap with the O/E like sites, indicating that they are distinct binding sites, and may bind to different types of proteins.

### Motif MV12 interacts with nuclear proteins from olfactory and vomeronasal sensory neurons

**Figure 3:**
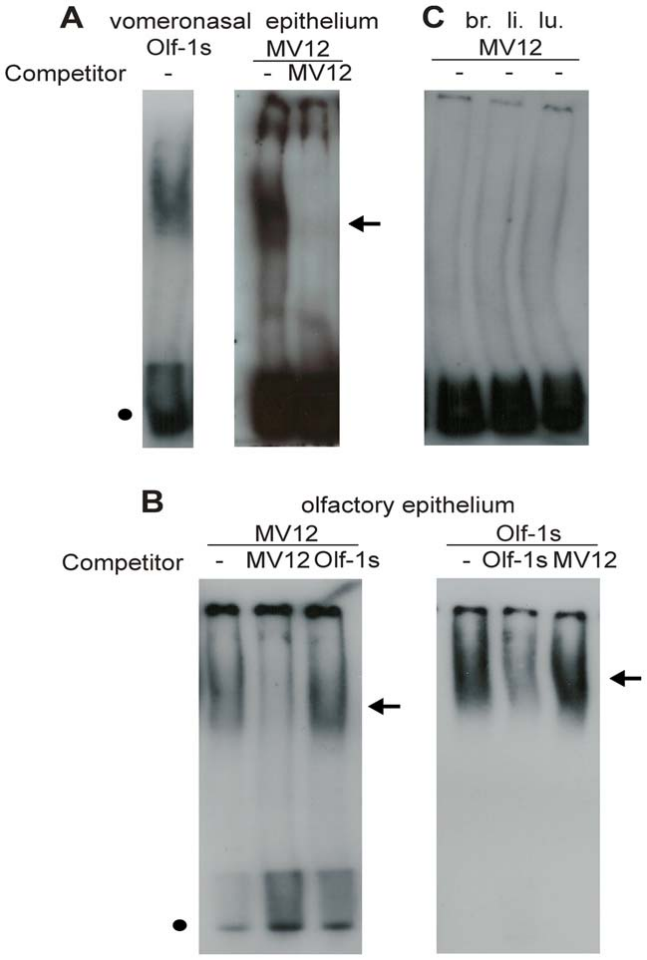
Binding of nuclear proteins to MV12. A labeled double-stranded oligonucleotide corresponding to motif MV12 was incubated with nuclear extracts from vomeronasal epithelium (A), olfactory epithelium (B), and brain (br.), liver (li.) or lung (lu.) (C). The Olf-1 binding site (*Olf-1s*) was used as a positive control. MV12 forms complexes with vomeronasal (A) and olfactory epithelia nuclear proteins (B). These complexes are competed by the addition of a 100-fold molar excess (competitor) of unlabeled MV12 oligonucleotide, but not by the addition of a 100-fold molar excess of unlabeled *Olf-1s* oligonucleotide (shown here only for the olfactory epithelium nuclear proteins, in B). The Olf-1s/olfactory epithelium protein complex is competed by the addition of a 100-fold molar excess of unlabeled *Olf-1s* oligonucleotide, but not by the addition of a 100-fold molar excess of unlabeled MV12 oligonucleotide (B). Complexes are indicated by arrows. The positions of the free probes are indicated by filled circles.

We next performed gel shift assays to determine whether the motifs shown in Figure 2A are able to form complexes with nuclear proteins extracted from the vomeronasal epithelium. As expected, the binding site for Olf-1 (*Olf-1s*) forms a complex with vomeronasal nuclear proteins, probably with the O/E like proteins that are expressed in the VNO (Fig. 3A). A weaker (based on the fact that longer film exposure times were required to detect it), but specific complex was also observed for MV12 (Fig. 3A), but not for the other motifs (not shown). Formation of the MV12 complex was inhibited by pre-incubation of the binding reaction with a 100-fold excess of the corresponding specific (MV12) unlabeled oligonucleotide (Fig. 3A), but not by pre-incubation with the unlabeled Olf-1 binding site (*Olf-1s*) (not shown). MV12 also formed a complex when incubated with olfactory epithelium nuclear proteins (Fig. 3B). Formation of this complex was also inhibited when the binding reaction was pre-incubated with the unlabeled specific oligonucleotide, but not by pre-incubation with *Olf-1s* (Fig. 3B). Conversely, *Olf-1s* forms a complex with olfactory nuclear proteins, which is not inhibited by pre-incubation with the MV12 unlabeled oligonucleotide (Fig. 3B). No MV12 complexes were formed when nuclear protein extracts from brain, liver or lung were used (Fig. 3C).

Altogether, these results show that motif MV12 is able to interact with proteins that are present in the nuclei of both vomeronasal and olfactory sensory neurons, but not in nuclei from other unrelated tissues, such as brain, lung and liver. In addition, they show that these proteins are not O/E like proteins, but must be different types of DNA binding proteins. The determination of the identity of the proteins involved in this interaction should contribute to the understanding of the role played by MV12 and related motifs in OR/V1R gene regulation.

### Motifs resemble binding sites for transcription repressors

**Figure 4:**
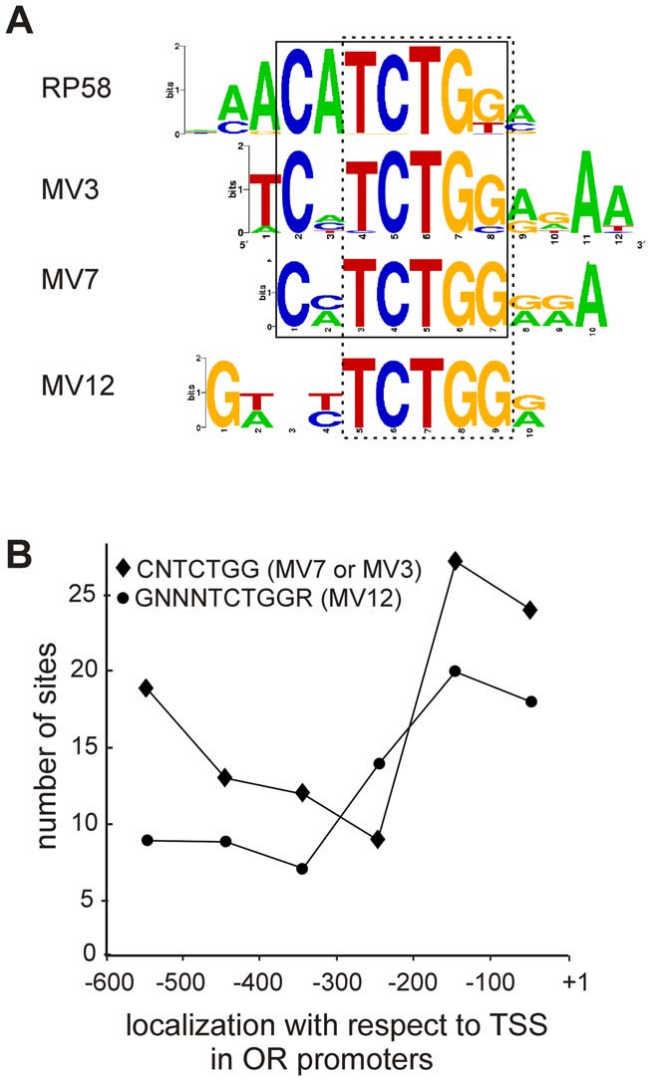
Alignment of MV3, MV7 and MV12 motif sequences and their matching transcription factor binding site. A) The output of the STAMP program is shown. The MV3, MV7 and MV12 consensus sequences are aligned, together with the binding site for RP58. The consensus sequence that is common to MV3, MV7 and RP58 (CNTCTGG) is boxed. The consensus sequence TCTGG common to MV3, MV7, MV12 and RP58 is also indicated (dashed line box). B) Localization of the V1R promoter motifs with respect to the transcription start sites of the OR genes. The number of sites for the V1R consensus sequences CNTCTGG and GNNNTCTGGR (corresponding to MV12) found across the OR promoter regions are shown as indicated. The positions of the TSS (+1) and 600 bp upstream of the TSS (−100 to −600) are indicated.

We next used the program STAMP (http://www.benoslab.pitt.edu/stamp/) to search databases for DNA motifs that match the V1R promoter motifs. We found a known transcription factor binding site that is highly similar to the MV3 and MV7 motifs, and similar to MV12. Alignment of these motifs shows a conserved consensus sequence CNTCTGG (as shown in Fig. 4A) which resembles the motif CH+BTB-POZ_RP58_M00532 in the TRANSFAC v11.3 database. RP58 (Repressor Protein with a predicted molecular mass of 58 kDa) belongs to the BTB-ZF (BTB-Zinc finger) or POK (POZ and Krüppel) family of transcription factors and contains both a Krüppel-like zinc finger DNA binding domain and a BTB domain [43], [44]. The BTB (for *Broad Complex*, *tramtrack* and *bric à brac*) also known as POZ (for poxviruses and zinc finger) domain is a conserved protein-protein interaction domain that can form multimeric aggregates [45]. BTB-ZF proteins are usually found in multiprotein transcription repression complexes, and are involved in transcriptional regulation through chromatin remodelling [43], [44]. The RP58 protein is a transcriptional repressor and was found to be associated with condensed chromatin regions in the nucleus [46]. A novel transcriptional repressor that shows high amino acid sequence identity to RP58 (named simiRP58) has been recently identified [47]. It is likely that simiRP58 binds to the same DNA motifs as RP58, since their zinc finger domains are highly conserved.

### Analysis of OR promoter regions

We next asked whether OR gene promoter regions contain the same motifs found in the V1R gene promoter regions. The promoter regions (600 bp long) of the 198 OR genes that we had previously determined [36] were searched for the presence of the V1R promoter motifs. Even though the CNTCTGG and TCTGG consensus sequences are present in higher percentages of the VNO promoter sequences (77% and 97% respectively), they were found respectively in 76 (38%) and 164 (82%) out of the 198 OR genes. A consensus sequence for the MV12 motif, GNNNTCTGGR (where R = G or A), was found in 79 (40%), and the consensus sequence TCTGGR common to MV3, MV7 and MV12 was found in 140 (71%) of the OR genes. The consensus sequences CNTCTGG and GNNNTCTGGR are concentrated near the TSSs (between +1 and −300 bp) of the OR genes (Fig. 4B). In order to determine whether these percentages are significant, we compared them to the percentages of promoters bearing the same motifs in a random selection of mouse gene promoters. The complete set of mouse promoters (containing 20.276 promoters) was retrieved from the UCSC Genome Browser (http://genome.ucsc.edu; file upstream1000.fa) and searched for the consensus sequences. The CNTCTGG motif was found in 22.6% of these sequences (p-value = 0.0001 (chi-square = 14.94; d.f. = 1)), the TCTGG motif in 62% (p-value = 0.0069 (chi-square = 7.31; d.f. = 1)) and the GNNNTCTGGR motif was found in 23% (p-value<0.0001 (chi-square = 16.01; d.f. = 1)) of these promoter sequences, showing that the motifs are significantly enriched in the OR promoter sequences. For the remaining motif MV15, only a small number of sites were found in the OR promoter regions. Interestingly, the shorter consensus sequence TCTCYCYY (where Y = C or T) within MV15 was found in respectively 82% and 52% of the V1R and OR gene promoters.

### Expression of BTB-ZF genes in the olfactory and vomeronasal epithelia

It is known that the mouse genome contains more than 40 BTB-ZF genes [48] and many of them show Zinc finger domains that are similar to that of RP58. We next examined which members of the BTB-ZF gene family are expressed in the olfactory epithelium. To do this, degenerate PCR primers were designed to be targeted to conserved sequences of the mouse BTB domains. Four degenerate primer combinations were used with olfactory epithelium cDNA in PCR reactions, and only products with the expected sizes (150 bps) were obtained. These products were subcloned and sequenced. The majority of the sequences correspond to BTB-ZF genes, while some correspond to other proteins that have a BTB domain but no Zinc finger domains (not shown). Among the identified sequences there are five different BTB-ZF genes (Table 2). The vast majority of the sequences correspond to Zbtb7b, while only a few clones correspond to Zbtb44, Zbtb7a, Zbtb-contig13 (an unannotated BTB-ZF gene present within a mouse chromosome 13 contig) and RP58.

**Table 2:** Expression of BTB-ZF genes in the olfactory epithelium identified by degenerate RT-PCR.

BTB-ZF gene	Also known as	Gene ID	Number of clones
Zbtb7b	Thpok; Zfp67; c-Krox	22724	100
Zbtb-contig13	Unannotated gene	NT_039578.7	3
Zbtb44	Btbd15	235132	2
Zbtb7a	Zbtb7; Pokemon, Lrf	16969	1
Zfp238	RP58; Znf238	30928	1

Zbtb-contig13 corresponds to an unannotated BTB-ZF gene contained within a mouse chromosome 13 genomic contig. Number of clones denotes the number of sequences obtained for each BTB-ZF out of a total 181 sequenced clones.

We also analyzed which BTB-ZF genes are preferentially expressed in the VNO epithelium. The same degenerate primers were used with VNO cDNA in PCR reactions, and six different BTB-ZF genes were identified (Table 3). Five of these are the same that were identified in the OE, however, the number of clones obtained for each one of these genes varied: while the majority of clones corresponded to Zbtb7b in the OE, only a few corresponded to this same gene in the VNO. Conversely, while the majority of the clones corresponded to RP58 in the VNO, only one clone corresponded to this gene in the OE.

**Table 3:** Expression of BTB-ZF genes in the vomeronasal epithelium identified by degenerate RT-PCR.

BTB-ZF gene	Also known as	Gene ID	Number of clones
Zfp238	RP58; Znf238	30928	17
Zbtb-contig13	Unannotated gene	NT_039578.7	10
Zbtb7a	Zbtb7; Pokemon, Lrf	16969	7
Zbtb7b	Thpok; Zfp67; c-Krox	22724	6
Zbtb44	Btbd15	235132	2
Zbtb20	Zfp288, DPZF, Oda8	56490	1

Zbtb-contig13 corresponds to an unannotated BTB-ZF gene contained within a mouse chromosome 13 genomic contig. Number of clones denotes the number of sequences obtained for each BTB-ZF out of a total 69 sequenced clones.

### Tissue distribution of the BTB-ZF genes expression

**Figure 5:**
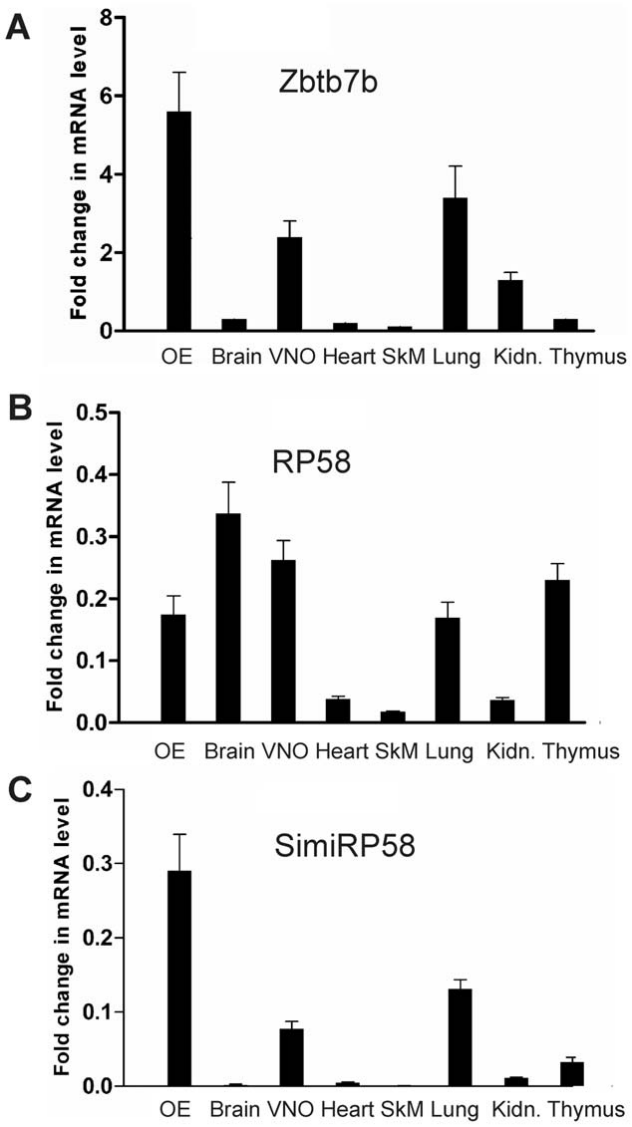
Relative gene expression levels of Zbtb7b, RP58 and *simiRP58* across different mouse tissues. Quantitative RT-PCR was performed in triplicate using primers specific for Zbtb7b, RP58, *simiRP58* and GAPDH with cDNAs prepared from mouse olfactory epithelium (OE), brain, vomeronasal organ (VNO), heart, skeletal muscle (SkM), lung, kidney and thymus. The levels of Zbtb7b, Rp58 and *simiRP58* mRNAs detected in each tissue were normalized to GAPDH and are shown relative to the expression levels in testis.

We next used real-time quantitative PCR analysis to quantify the relative expression levels of the Zbtb7b, RP58 and simiRP58 genes across nine mouse tissues (Figure 5). The Zbtb7b gene was expressed at higher levels in the olfactory epithelium, and at lower levels in the lung, VNO and testis (Figure 5). The simiRP58 gene showed a similar pattern of expression, except that the highest levels of expression are in testis and not in the olfactory epithelium (Fig. 5). Differently from Zbtb7b and simiRP58, RP58 shows a less restricted distribution of gene expression: it is expressed at equivalent levels in six out of the nine tissues examined (olfactory epithelium, brain, VNO, lung, thymus and testis) (Fig. 5). Altogether these results show that the Zbtb7b gene expression is preferentially expressed in the olfactory epithelium. They also show that RP58 and simiRP58 are expressed at higher levels in testis and at much lower levels in the OE and VNO.

### Predominant expression of Zbtb7b in olfactory sensory neurons

**Figure 6:**
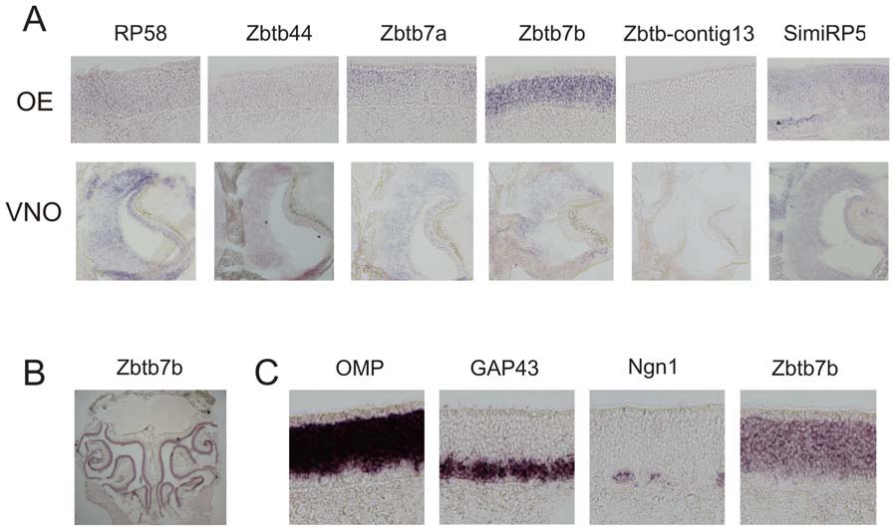
Expression pattern of the BTB-ZF genes in the olfactory and vomeronasal epithelia. (A) Coronal sections through the olfactory epithelium (OE) or vomeronasal epithelium (VNO) were hybridized with antisense digoxigenin-labeled probes specific for Zbtb7b, Zbtb-contig13, Zbtb44, Zbtb7a, RP58 or simiRP58 as indicated. Corresponding sense digoxigenin-labeled probes were used as negative controls (not shown). B) Zbtb7b is expressed throughout the olfactory epithelium. C) Zbtb7b is expressed in the neuronal layers of the olfactory epithelium (mature and immature neurons). OMP, GAP43 and Ngn1, used as a control, are respectively expressed in mature olfactory neurons, immature olfactory neurons and neuronal progenitors.

We performed *in situ* hybridization experiments to analyze the expression of the identified BTB-ZF genes in the olfactory epithelium. Among all of the BTB-ZF genes analyzed, Zbtb7b is the one that shows the strongest hybridization signals in the olfactory sensory neurons (Fig. 6A and B). The other BTB-ZF genes show much weaker hybridization signals, except for Zbtb7a and simiRP58 which show intermediate hybridization signals (Fig. 6A). Also, the Zbtb7b expression pattern is confined to the olfactory sensory neurons (Fig. 6B), while the other genes show a more ubiquitous pattern of expression, specially RP58 and simiRP58 (not shown). Comparison of adjacent sections hybridized with probes that label immature neurons (GAP43), mature neurons (OMP) and progenitors (neurogenin 1), shows that Zbtb7b is expressed in both the neuronal cell layers but not in the basal cell layer (Fig. 6C).

We also analyzed if the BTB-ZF genes are expressed in the vomeronasal epithelium. Except for contig 13, all of the analyzed BTB-ZF genes showed weak to moderate expression in the VNO (Fig. 6A).

Altogether these results show that Zbtb7b is the predominant BTB-ZF in the olfactory neurons and it is likely to play a role in gene regulation in these neurons. In order to determine whether Zbtb7b is involved in OR gene regulation we analyzed the expression pattern of OR genes in mice that are knocked out for Zbtb7b [49]. These mice have the entire coding region from the Zbtb7b gene disrupted, and therefore do not express the Zbtb7b protein [49]. The expression of 19 different OR genes in the olfactory epithelia from heterozygous (+/−) and homozygous (−/−) Zbtb7b knocked out mice was analyzed using *in situ* hybridization. Four of these OR genes belong to class I (MOR1-1. MOR15-1, MOR25-1 and MOR33-1) and 15 belong to class II (MORs 138-3, 178-1, 180-1, 184-1, 185-1, 189-1, 203-1, 244-3, 260-1, 271-1, 277-1, 284-2, 171-2, 104-1 and 139-1). We observed the typical zonal punctate staining pattern in the olfactory epithelium for all of these ORs, with no obvious differences between the heterozygous (+/−) and homozygous (−/−) mice (data not shown). The absence of an evident phenotype in Zbtb7b knocked out mice could be due to the presence of related genes which could be involved in the same regulatory network. For example, the Zbtb-contig13 (unannotated) gene, which we have also identified in the degenerate RT-PCR screening (Tables 2 and 3), shows a highly similar amino acid sequence to the Zbtb7b protein (∼83% amino acid sequence identity). Whether this gene, or another member of the BTB-ZF family, is required for OR gene regulation still remains to be determined.

## Discussion

The fact that both olfactory and vomeronasal sensory neurons express only one receptor gene from a large number of possible choices suggests that a similar mechanism is used to regulate respectively OR and V1R gene transcription in these neurons. In the present study we analyzed whether OR and V1R gene promoter regions share DNA elements which could be involved in this common mechanism of regulation. We first determined the promoter regions for 39 V1R genes by using RLM-RACE. These promoter regions should contain elements that are required for V1R gene expression, however, it is important to point out that we have not analyzed whether they are sufficient for correct V1R gene expression. We then searched these promoters for common elements and identified motifs that are present in 49–74% of the promoters and that are concentrated near the TSSs of the corresponding V1R genes. Two of these motifs share a consensus sequence (CNTCTGG) that is also present in the promoter regions of ∼40% of the 198 OR genes that we analyzed, and are also concentrated near the TSSs of the corresponding OR genes. This sequence matches closely to the sequence CATCTGG which is the binding site of the transcriptional repressor RP58, which belongs to the BTB-ZF family [46]. RP58 was shown to be associated with condensed chromatin regions in the nucleus of IMR32 (human neuroblastoma) cells, indicating that its transcriptional repression activity may be important in regulating heterochromatin-mediated gene inactivation processes [46]. Accordingly, it was shown that Dnmt3a, which associates with histone deacetylase HDAC1, is recruited by RP58 to specific regulatory sites [50]. It has been recently reported that homozygous *RP58* ^−/−^ mice die shortly after birth, but the cause of the death remains unknown [51]. These mice show abnormal development of certain brain regions, but it is still unknown whether their olfactory system is also perturbed.

We found that Zbtb7b is the member of the BTB-ZF family that shows the highest level of expression in the olfactory epithelium. Zbtb7b, also known as Th-POK (for T-helper-inducing POZ/Krüppel factor), was shown to be essential for CD4 cell fate decision during T lymphocyte development [52], [53] and it was first identified as the gene that is mutated in a spontaneous mouse mutant, termed ‘helper deficient’ (HD), which lacked CD4 cells [54], [55]. It has been shown that Zbtb7b (ThPOK) is expressed in CD4 cells (but not in CD8 cells) where it prevents the expression of CD8 lineage genes, thereby promoting CD4 lineage commitment [53]. Zbtb7b mutant mice do not show altered OR gene expression in the olfactory epithelium, at least for the OR genes that we have analyzed, however, it still needs to be determined whether another related member of the BTB-ZF family is involved in OR gene regulation.

### Repressors and OR/V1R gene regulation

So far, only transcriptional activators, such as Lhx2, Emx2 and Olf-1 have been implicated in OR gene expression. Lhx2, a LIM-homeodomain protein, was shown to bind to the MOR71 promoter region [56]. *Lhx2* deficient mice lack mature olfactory sensory neurons, indicating that this homeodomain protein is required for olfactory sensory neuron development [56], [57]. In these mutant mice the expression of class II OR genes is abolished, while most class I OR genes are still expressed in a few OMP positive neurons located in the dorsal region (corresponding to zone 1) of the olfactory epithelium [58]. These results indicate that Lhx2 is directly involved in class II OR gene expression, but is not required for class I OR gene expression. The Emx2 homeobox transcription factor was also shown to bind to the mouse OR71 gene promoter [56]. *Emx2* mutant mice develop a normal olfactory epithelium, except that they have a reduced number of mature olfactory sensory neurons [59]. The expression of many OR genes is reduced greater than a 42% reduction in mature olfactory sensory neurons, indicating that the absence of Emx2 is not altering OR gene expression only because of a general defect in olfactory sensory neuron development. Emx2 seems to act directly on OR gene promoters to regulate gene transcription. As described above, the precise role played by Olf-1 in OR gene expression is still unclear. We found that V1R genes also have O/E like sites in their promoter regions, but their role in V1R gene regulation is also unclear.

Our results suggest that transcriptional repressors bind to the promoter regions of OR and V1R genes. Nevertheless, it is important to point out that additional experiments should be performed in the future to determine whether the motifs identified in this study are able to bind repressors and thereby repress the transcription of these genes. What could be the role played by repressors in OR/V1R gene regulation? It makes sense that in order to achieve the one neuron-one receptor rule it is necessary that the expression of all OR/V1R genes (except for one single chosen gene) is tightly repressed in the sensory neurons. Recently, Magklara and colleagues [60] demonstrated that OR genes are marked with the hallmarks of constitutive heterochromatin, H3K9me3 and H4K20me3, in the nuclei of olfactory neurons, but not in nuclei from liver cells. It is possible that through interaction with transcription repressors like the BTB-ZF or others, these and other types of silencing epigenetic marks are added to the OR and V1R genes, so that they are localized to nuclear compartments that lack the transcription machinery or that bear an excess of inhibitory factors. In this case, OR/V1R gene expression would not depend on activation of a single chosen gene, but would depend on the release of a single gene from the default repressive state. Interestingly, Magklara and colleagues [60] also showed that the repressive heterochromatic mark (H3K9me3) is absent from one transcriptionally active OR gene allele. The study of the regulatory proteins involved in transcriptional repression and of the mechanisms that alleviate the repression for one chosen gene should contribute to the understanding of OR/V1R gene choice regulation.

## Materials and Methods

### Animal Procedures

All procedures undertaken in this study were approved by the University of São Paulo Chemistry Institute's Animal Care and Use Committee, under the protocol number 1/2010.

### 5′ RNA Ligase Mediated Rapid Amplification of cDNA Ends

Total RNA was purified from C57BL/6J mice (6–7 weeks old) vomeronasal epithelium using TRIzol reagent (Invitrogen), following the manufacturer's instructions. RLM-RACE was performed exactly as previously described [36].

Twenty-five-microliter PCR reactions containing 1 µL of RLM-RACE cDNA, 0.2 mM dNTP, 1.5 mM MgCl_2_, 0.5 µM forward primer (GeneRacer 5′ primer), 0.5 µM reverse primer (0.5 µM subfamily specific or 2 µM degenerate primers) and 1.25 U of Platinum Taq DNA polymerase (Invitrogen) were heated to 95°C for 2 min, followed by 25 thermal cycles of 95°C for 1 min, 50°C for 3 min, 72°C for 2 min, and a final incubation at 72°C for 10 min.

Twenty-five-microliter nested PCR reactions were done using 1 µl of a 200-fold dilution of the primary PCR product and 30 cycles as above.

The following degenerate primers, matching to V1R TM3 regions, were used:

VNR: GGI(C/G)(A/T)IA(T/G)IGTGA(C/T)IGC(C/T)TGIA(A/C)IAC(A/G)CTIAG

VNR nested: A(C/G)(A/G)CAIGTIG(A/T)(A/G)CAIA(T/G)IGAI(A/G)(T/G)IC(C/T)IC(T/G)

The following subfamily specific primers were used:

V1RA: GCCTGAGATCCCAATGCC

V1RA nested: GAGACCAATGGGCAGGTC

VR1B: CAGCTATGAGTCCCATAG

V1RB nested: CCAAAGGACATTCAGCAG

V1RD: GTATGCAAGTTTACATTTG

V1RD nested: GTTATTTAAGACACTGAAG

V1RE: GAAATTTCCCAGAATTCC

V1RE nested: GGTGCATGAGAATCAAATC

V1RF: GAATCCAGTCTATAGAC

V1RF nested: CAATTTGCATCCAATATC

V1RG: GAATTGCCAAGCATTCCC

V1RG nested: GTCAAGTGCTCTATAATC

V1RJ: GATTGTCAACATATTGAC

V1RJ nested: CTGGGAGTGATGGTGATG

V1RL: CCAGTGAAGTCAGCGATG

V1RL nested: GACTTGGAATTGTTGAGG

### Cloning and sequencing

The RLM-RACE PCR products were cloned into the pCRII vector (Invitrogen). Colonies containing V1R cDNAs were selected by colony PCR using T7 or SP6 primers plus the V1R degenerate or specific primers. Plasmid DNA purification and sequencing was performed as previously described [36]. All cDNA sequences were deposited in Gen Bank under the accession numbers: EX601137–EX601479.

### Sequence analysis

cDNA sequences were aligned to the mouse genome sequence using BLAT [39] and Sim4 [40] as described before [36]. Only the Sim4 alignments showing average percent identity ≥93%, entire sequence alignement >55%, and with the best score (based on the nucleotide identity over the entire alignment) were selected. The cDNA sequences were clustered based on their genomic coordinates.

A FASTA sequence file containing the 39 V1R promoter sequences (1 kb upstream of the TSS, Figure S2) was analyzed using the Gibbs Recursive Sampler (http://bayesweb.wadsworth.org/cgi-bin/gibbs.8.pl?data_type=DNA) (motif lengths used were 8,8,6,8,8 and 10,10,8,10,10, and the program's default background settings for mammals were used) and Consensus (http://bifrost.wustl.edu/consensus/html/Html/interface.html) (motif widths were set to 6,6,6,6; 8,8,8,8 and 10,10,10,10). The location of the motifs within the promoter regions was determined using Siteseer (http://www.chick.manchester.ac.uk/SiteSeer/). The STAMP tool (http://www.benoslab.pitt.edu/stamp/) was used to search for similarities between input motifs and a database of annotated motifs. The motif degenerate sequences that were used for the analysis are shown in the Figure S3.

### Gel shift assay

Gel shift assays were performed using the digoxigenin gel shift kit (Roche Applied Science) as described in [36]. Nuclear extracts were prepared from olfactory epithelium or from VNO dissected from C57BL/6J mice [36]. Brain and liver control nuclear extracts were checked for the presence of DNA binding proteins by using the TFIID binding site in gel shift assays (Figure S4). Binding reactions contained 22 µg (vomeronasal epithelium) or 30 µg (olfactory epithelium) of nuclear protein extracts and 11 µg or 2 µg respectively of poly[d(I-C)]. Films were exposed for 40–60 minutes to detect the protein –MV12 complexes, and 15 (olfactory epithelium) to 30 (vomeronasal epithelium) minutes to detect the Olf-1 s/protein complexes. The following pair of complimentary oligonucleotides was used as double-stranded DNA probe for the gel shift reactions (motif sequence is underlined):

MV12 CACACGGAAACCCAGAGCTCACCTGTCACTA and AGTGACAGGTGAGCTCTGGGTTTCCGTGTGT;

### Quantitative RT-PCR

RNA was prepared from different mouse tissues using TRIzol reagent (Invitrogen). cDNA was synthesized from 1 µg of total RNA which was previously treated with RQ1 RNAse free DNase (Promega). First, 1 µg of total RNA plus 100 ng of oligo (dT) in 13.5 µl of DEPC treated water were incubated for 2 minutes at 70°C. The reaction was rapidly chilled on ice and used to synthesize cDNA in 20 µl of 1 X Superscript II first strand buffer containing 0.5 mM dNTP, 3 mM MgCl_2_, 20 U RNAse inhibitor (RNaseOUT, Invitrogen) and 200 U of Superscript II reverse transcriptase at 42°C for 60 minutes. The product was diluted to 100 µl with DEPC treated water. Zbtb7b, RP58, simiRP58 and GAPDH (glyceraldehyde-3-phosphate dehydrogenase) were quantified by real-time PCR using the ABI (USA) 7300 Real-Time PCR system. Primer sequences for Zbtb7b were TAGGCAAGGCAACTCACTTG (forward) and ATCAGAAGAGGCTATGTACAG (reverse). Primer sequences for RP58 were CTGCAAATTGATAACTTCGCTAC (forward) and CTCTGAGCTGGCATGGGTTC (reverse). Primer sequences for simiRP58 were ACGGAGTGGGATTTGGACA (forward) and CAGCCCCATGAAGCCAGTAG (reverse). Primer sequences for GAPDH were AAATGGTGAAGGTCGGTGTG (forward) and TGAAGGGGTCGTTGATGG (reverse). All reactions were performed by using a standard real time PCR protocol (1 cycle of 95°C for 10 min, 40 cycles of 95°C for 15 s, and 60°C for 1 min). Data was normalized by using GAPDH as reference. Relative gene expression across tissues was then calculated as 2 ^−ΔΔCt^, using the sample of testis as calibrator, according to [61]. Each reaction was performed in triplicate and the standard deviation was inferior to 0.3.

### Degenerate RT-PCR

The amino acid sequences of the BTB domains of all of the BTB-ZF proteins in the BTB domain database (http://btb.uhnres.utoronto.ca/, [48]) were aligned and browsed for conserved sequences. Two forward and two reverse degenerate primers were designed to match respectively the conserved H(KR)(ANV)VLAA(CSF)S and (LI)L(DE)(FY)(MAI)Y(TS)(GA)(KRT) amino acid sequences. The primer sequences are: BTBF1: CA(CT)AA(GA)G(TC)IGTICTIGCIGCIT(TG)(CT)(AT); BTBF2: CA(CT)(AC)GIG(TC)IGTICTIGCIGCIT(TG)(CT)(AT); BTBR1: (TG)I(GC)CIG(AT)(GA)TAIAT(GA)(TA)AITCIA(AG)IA(AG) and BTBR2: (TG)I(GC)CIG(AT)(GA)TAIGC(GA)(TA)AITCIA(AG)IA(AG).

Degenerate PCR reactions were performed as described above but using cDNA prepared from olfactory epithelium or vomeronasal epithelium using random primers instead of oligo dT, and 2 µM final of each one of the primers (forward and reverse). Reactions were heated at 95°C for 2 minutes, followed by 40 cycles of 95°C for 1 minute, 50°C for 3 minutes and 72°C for 2 minutes and a final incubation at 72°C for 10 minutes. PCR products showing the expected size (∼150 bp) were obtained for the BTBF1/BTBR2, BTBF2/BTBR1 and BTBF2/BTBR2 pairs of primers for the olfactory epithelium. These products were purified from agarose gels and subcloned into the pCRII vector (Invitrogen). Plasmid DNA from colonies was prepared and sequenced as described in [36].

### *In situ* hybridization

*In situ* hybridization was performed according to [62]. Noses were dissected respectively from 3 weeks old mice and freshly embedded in Tissue-Tek O.C.T. compound (Sakura Finetek USA Inc.). Sequential 14–16 µm sections were prepared with a cryostat and hybridized with digoxigenin-labeled cRNA probes prepared from Zbtb7b (NM_009565.4); Zbtb-contig13 (within NT_039578.7); Zbtb44 (NM_172765.2); Zbtb7a (NM_010731.3); RP58 (AF140224) and simiRP58 (BC05108) sequences using the following primers: Zbtb7b: GTTGGTATCAGAATCGAACG (forward) and ATGACTCAGCGTTGGAACTG (reverse); Zbtb7a: CGAGTAAGCAGTTCTCTGTC (forward) and CTGATCTTGGCTATGTCCAC (reverse); Zbtb44: ATGCTCAGAGTTCATGAAGTC (forward) and GACTGTGGACTGATCAGAAG (reverse); Zbtb-contig13: CACAGTTGGAATCCTAGATC (forward) and CTGAATACCTAGAGCATACAC (reverse); RP58: CATCCTGCAGATCCACCTGAG (forward) and TAAGGTCCAGTCTCTGACAGTG (reverse); simiRP58: ACGGAGTGGGACTTTGGACA (forward) and CACTCAGTCACAGGGTCCTAC (reverse). Except for Zbtb44 and RP58, the probes were prepared from the 3′ UTR sequences of the cDNAs in order to avoid cross-hybridization. The probes for GAP43 (U63841) and Ngn1 (NM_008083.2) were prepared using the following primers: GAP43: ACCATGCTGTGCTGTATGAG (forward) and gttcaggcatgttcttggtc (reverse); Ngn1: GTTCAGGCATGTTCTTGGTC (forward) and tacaaaggcctagtggtatg (reverse).

### Genotyping of Zbtb7b (ThPOK) mice

The 450 bp wild type allele was amplified by using primers TGGGGTGATCACCCAAGGAG and CACCCCAGTCTTTCAAGCCT.

The 550 bp targeted allele was amplified by primers TGAACCGCAGTTCCCTTGTGC and CACCCCAGTCTTTCAAGCCT. The amplification conditions for both pair of primers were: 95°C for 2 min, and 30 cycles of 95°C for 30 sec, 58°C for 30 sec, 72°C for 30 sec, followed by 10 min at 72°C.

## Supporting Information

Figure S1 **The 5′ structure of V1R genes (schematic representation of the 5′ regions of the 39 V1R genes analyzed in this study).** The 5′ structures of the 39 genes analyzed in this study are shown. The previously annotated V1R ORFs [13] are represented as grey boxes. The cDNA exons are represented as black boxes and the introns by black lines. The sizes of the introns that are not shown to scale, are indicated in kilobases.
(PDF)

Figure S2 **V1R promoter sequences.** FASTA file containing the 39 V1R gene promoters.
(PDF)

Figure S3 **Motifs consensus sequences.** The consensus sequences of the V1R motifs that were used in the bioinformatics analysis are shown.
(PDF)

Figure S4 **Control experiment with the nuclear protein extracts.** Labeled double-stranded oligonucleotides corresponding to TFIID binding site or motif MV12 were incubated with nuclear extracts prepared from brain or liver. The TFIID motif forms complexes with proteins from brain and liver, while the MV12 motif does not.
(PDF)
