# Supplementary figures and images for: Common Promoter Elements in Odorant and Vomeronasal Receptor Genes
Source: PLoS One. 2011 Dec 28;6(12):e29065. doi: 10.1371/journal.pone.0029065 (PMC3247230; doi:10.1371/journal.pone.0029065)

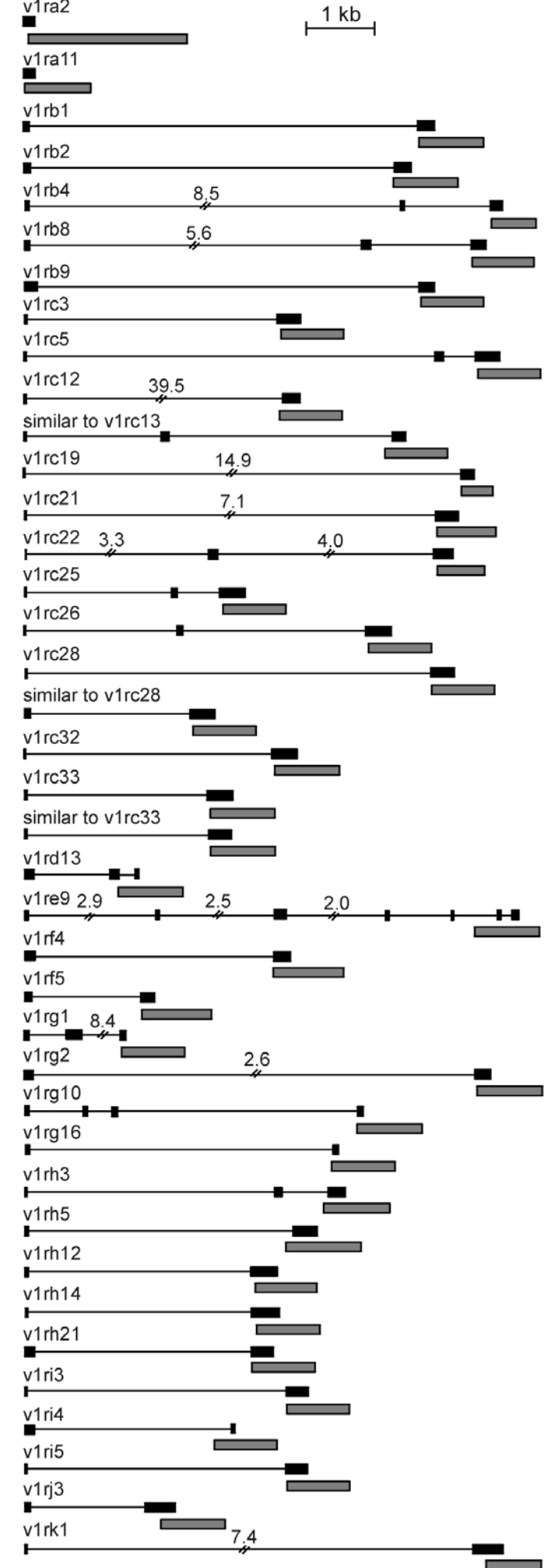

Supplemental Figure 1

Supplement: Figure S1 — The 5′ structure of V1R genes (schematic representation of the 5′ regions of the 39 V1R genes analyzed in this study). The 5′ structures of the 39 genes analyzed in this study are shown. The previously annotated V1R ORFs [13] are represented as grey boxes. The cDNA exons are represented as black boxes and the introns by black lines. The sizes of the introns that are not shown to scale, are indicated in kilobases. (PDF) [file pone.0029065.s001.pdf]

1 2 3 4

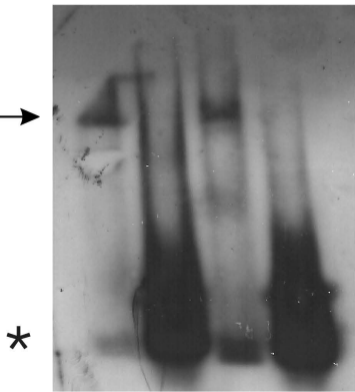

1- Brain/TFIID

2- Brain/ MV12

3- Liver/TFIID

4- Liver/MV12

Supplement: Figure S4 — Control experiment with the nuclear protein extracts. Labeled double-stranded oligonucleotides corresponding to TFIID binding site or motif MV12 were incubated with nuclear extracts prepared from brain or liver. The TFIID motif forms complexes with proteins from brain and liver, while the MV12 motif does not. (PDF) [file pone.0029065.s004.pdf]
