# Supplementary material for: Common Promoter Elements in Odorant and Vomeronasal Receptor Genes
Source: PLoS One. 2011 Dec 28;6(12):e29065. doi: 10.1371/journal.pone.0029065 (PMC3247230; doi:10.1371/journal.pone.0029065)
Supplement: Figure S2 — V1R promoter sequences. FASTA file containing the 39 V1R gene promoters. (PDF) [file pone.0029065.s002.pdf]

>V1rh21
AGTCCAAACAAATGCTCCTCTCAACTGGTTAAAAAATAGAAATTAGAAAA
CCTGTGTCTTTCTGTGCAGTCTCTCCATAATGAAAATGCTTTGGAAAGTC
AAGGCAGGAGAGAATAAAGTGCAGTATTCTCAATACCAGTGACAAAGAAT
GTTTGTAAGACATATCTCTAGGGTTGATTCCAGGTTCAGTAAGAAAATGC
AATGTATTGAAACCAAATTTTACAAATATCTCAAAAGGGACAAATATATT
TTATATACAATAAATGAGGAAAATACTTTAAAGAAGAGAAATACATTAAT
CCTGCCCATTTGCTTCAGGGAAAATTAGGGTTTAATTTTTATATTTGTAT
TACCCCTTGAAAATATGATGGGCCAACTTGATCAACCTAGGATTCAATTT
TCCACTCAAAGCAAGGATAGAAGCAGTAAAGGGATTTTCTTCATTTACAG
CCCAAGTGTGTATTGTGCATAGAGTATTGGGAGTGCCTTTGAGTTACAAG
GAAACAGTAGTTAAAGACAGAATTTGAACTGAAAAAGGGAGGCTCATCTG
GCTTGAAGATACCATTTATTTAACAAAGAAGAAAGAAAGCATCACTTCTA
GTTGACTGATCTAGGTTGTGTCCAGATGTAGCAAAGACACCTTGTTTGCT
CAGATGTGTGGTTGGAATGAGCTCAGAAAGTATGGTGTAGAGTGTTGGGT
ATAATTTATCATGTCCTCTACAGAAAGAAGGACTTGAGAAAGAATGGATC
TCTTTCTGTATCAGAAGCAGGACTCTATTCTGATCAATACATTTGGTTAA
GCAGTGTAAAGAGAGGACCAGGAACATGGGGACTCTAGATATAGAAGACT
TACTGTTTTTCACTTTGTCCCTTCATATGTTTCTATCATACAATGAAAAT
GTAATTAATCCAGAGGCAACTCTTGAGGCAAACAAATGTAGTCATTGAGG
AGGGAGAGGGAAAGTCATCCTTTAGGGACTGAGGTTCCACAGAGGGCTTC
>V1ri3
CCATATAGATTTGATTTAAATGACAACTTTTTTTATGAATACACCCTTGG
AGCCTCCTTGTGTTCACACTACTATATTATACATGAAAATAACAAAATGA
TTGAAATGCTGAATAAAAGAAAAATGTTTTCAAAGTCAGTGATCTTATAA
AGATTTTAGAAAAGAGAAAGAAAGAGATAAATTCAGGATATAGACCATAA
AGACAGCAATCCATATTAGACATTCAAAAAAAAAAAAGAGGTATTGAAGA
AAAGAGAAACAAACAAGAGAAGAAATAAAGAACTCATCTGGGAAGGGAGT
GACTCCTCTGCATAAAGTCAGCACTGCCCTGCTGCTCTGCCCTAGACACC
ACACCTTAAGACTAGACACTGACCCTAGCTCTGTCCTGGACACCTGAAGA
CTAAACATTGATCCTAAGCTATTGAATATCTTTTCATCTTTTCTTCCTGA
TGTTACTGAGTTTTAGAGTTTTATTTTTTATTAGACACATTTGTTTTATT
AACATGTATTTTACCTGTATATATTTTGCATGCATATATGTCTGTGCATC
ATGGATATGATTAATAACTGTGAAAGCCAAAGGAGGGCATTAGATCCCCT
GAAACTGGAGTTACAGATGTTTGGGAATCACCAAGGGAGTGCTGAGAATC
CAATAGGGGCATTTGCAAAAGACTAATTGAACTTTGAAATGTTCAGAGAG
CAAAAGTTTCAGTATCTCAGAAGTTTCTCCCAATCACTTCCTGGCTTTGG
GGTAAAATGAGGAGGGAAGTCCCAGGATCCAGGAAACTCAGGCACTGTAA
TTACAACCAGAAACTTTCTGGCTCAGCAAATGACAGGGCACCAGAGAGAT
CACAGACCTGACATCAAGGATGGAGATTGCAGAAGGAGCCCTTTCCCCAC
GGAGAAGCCAAACTTCTAACAGTGACATCCTATTAGCAATGACAGTTGCT
GTGAGCCCAGGGCTCAAGGACAATAAACAGGACCAAGCTATGTTGTGGGG
>V1rh3
GACCCATTTAACGGTACAATTAAGAGCACTTGATAGTCAGGCAGAAGACT
CATGTTGGGTTTGTAGCACCCACATGTTGATTCACAACCTCCTATAACTC
TACTTTCAGAGGATCCCATGCTTTCTTACAACCTCTGTGTGGGAAGATGC
CCTTCCAGGCTTCAGAAAGAGAAACTTGGACAGGCACAAAATCTTTACCT
ACAGTCTGTCCTGCCTGAAAAATATGCCAGAGCAATGGTGGCAAAAGTAT
CAATGAAAAGTATCAACGAAATGACTCCTTTTTTTTAAAGACCAGTAGTA
TTGGGGTTACCCTAGGTCTGTGGACGCTAGTAAATCTAGTGAAAAAAGTC
TGCCCTTAAGTAAAACCAGACGCATTTTAGTTATTTTCCTAAGTTTTTGC
CTCCTCTTCTGTGTTCTTATCTGCAGTGTGTAATTTAGTCATCATTAGAG
AGGCTTCCCCCAGCTGCAGAGAAGAATACACAGCAGGATTTTAGGCCGAA
AGCAAATCTATATGGGAGGTCTCCATTAAATCCCTCACCTCAAAGGACAG
AGAATCTCTTGAAAGAGGAGGCAGAAACATTGTAAGAGCCAGAGAGGCCA
AAGGTCACCAGGGAAGCAGGCCCTCTGAGGCAACTAAGCAAGGAGCATAT
AAGCTCACCGAAACTGAAGCAGAAAGCACCAGGCTTAAATGAATATACAG
AAAGTCCTCTGGAGATGCGAGAAGCTCAGTACACAGAAACTTGGTTAGTT
GTTCACTTTCTCACCTCACAGTCTTCCTCTCAAAATAATTAAAGCCTAAT
TAAGCTGGAAATAACTTCTGAGCAAAACAGATGTTGCAACTGAGAGAGAG
AGAGAGAGAAAAGGAAACTGTACTTCAGGGACTGAAGCTCTGCAGTGGCG

CCTTCCTTCCCCCTGACACTGTGACAGCAGGAAACCTGATGGCAGCCAAG
AGCCCCACAGATCCAGACACTACAAATATGACCTGGCAAGTGTCACCTTC
>V1ri4
GTAGTTTGGGAAAGTCTATCCAGCTGATCTGAATGTGTTTCTGGGCAACA
GTAGGTCACTTTCTGCTTCATCGTTTTATTGCTTCATCCCATGTGGATTT
GATTTAAATGACTACATTCCTTTATAAATCCAGACTATTCAATAAAATAA
TGAAATGCTTAAAATACTGAACAAAAAAATAATTGTTTCATTGTTTTCAA
ATAACCAGTGATATAAAGAGAATTTGGATAAACAGAATGGAAGAAAATAC
AGTAAATTCAGGACTAAGACCAGAAAGTCAGCAGTCCATATTAGACATTC
GGCAAGAAAAAATGAGATTTTGAAGAAAAAGCAACAAGTAAAAGGAGAAA
TGAAAAAGCTCAGCCAGGAGGGCAGTTGAATCCTCTGCATAAAAGCATCG
GTCAGAGCAAAAAGCAGACACAATTTCCAAAAGTCTCCCTGCTGCTCTGC
CATGGACACCACACCATAAGATTAGACACTGATTCTGGCCTGTAATCATC
TATTGTTCTGGACATACTAACCTTATAAAAAAAGAAACTTTATTTTTACT
AGATTTGTTCCTTTTAATGTGTGTTTTACTTTTACATATTTTGCCTGCAT
ATATGTCTGTGCATCATGTGTGTACTTGATGCCCATGGAGGTCAGAAGAC
GGCATTAGGTCTCCTGGAGTTATATATGGTTGTAAACCACCATATGGGTG
CTTGGAATTGAACCTCTGTAGGAGCAACAAGTGCTTAACTACTGAGTCAT
TGCTTGAACCCCAGTGCTATTTAGAAGTCTTCTCAGTTTCTCAGAACTTT
CCCACTGAAGTTCCTGGCTTTGGGAGAGGGAAGAGGAAAAAGTCATATTC
TCGAGGAAACTCAAGGTACTGTTTGTCCTCACAGCCCTCCTTGACAACAC
AGGCACTGTAATTAGAACCAGAAAGTTTCTTGCTCAGCAAATGACAGGGC
ACCAGGGAGAGCAGAGAGCTGACATCTAGAATGGAGATTGCAGAAGGAGC
>V1rh5
TCAGGGCCCCATTAGCAATGACAGCACCCATACAACTCACTGAACATGGA
GGGACTGAGCTGGTACCTACATAGAGCCTTCATCCCTACTTCTAAAATGT
TTGATGTGGGAAGGTACTCTTTCAGCCCTCACGAAGAGAAACATGGATCC
CAACTCAACCGTAAAACCTTTGACTTAGAATCTGTCCTATCAGAAAATAG
CCCAGGACAATGGTGACACAGAACTTATGGGACTAGTCAACCAATGTAGA
TGTTACTTAAGGTCCCACTCCAGCAGAAGGAACCCATTCCCAAAACTGTA
ACTAAGAACTAGAGACTAGACAGCTCACAGACCTAAGGTAAAACCAAACA
CTACTAGTCTTCAAAATATACAGTTCAATAAAATGACTCCTAATGATATT
CTTCTATGCTCATAGATCAGTGTGTTATCGAGCCTTCATTAAAGAGGCTT
CCTCTGGCAGCAGATAGAACAGATGTAGAGCTCCACAGTCAAAAACTAAG
CAGAAAGGGAGTCTACCTGGGAGGAAATCACTCCCCTCAGGGCACTGGGG
ATCTTTTGGAAGAGGAGGCAGAAAGACTGTAAGAGCTAGAGAGGAAGGGG
AATATTCGGAGAAAAAGGACCGCTGAATCAACCACGTGAGGTACATATTA
GCCAAAAGAGGCTGAAGTAGCAAGTACAGAACCACAATGAGTGTCCACAG
GTCCTCTGGAGACTCAGGAAGCTCAGTACACAGAACTTGGTTAGTTGCTC
ATTTTCTCACCTCACATCCTTCCTCTCAAAATAGTTAAACCCTAATTAAG
CTGGAATAATTTCTGAGCAAAACAGATGCTGTAACTGAGGGAGAGAGGGA
AGCTGTACTTCAGGGACTGAGGCTCTGCAGTGGCCTCCCTTCCCCCTGAC
ACTGTGACAACATGAAACCTGAAGTCTGCAGTACCTGATGACAGCCAAGA
GCCCCACAGATCCAGACAGTACAAATATGACCTGGCAAGTGTCACCTTCC
>V1rh14
TGACATATATAATGTACAACTTAGTTTCTCTGTACCTGGATTCCCCTGTT
CAATAGAGGGTATAGAGATCCCTGCGTCCTCAAGAGCTCTCATTTATATT
GCAGAAGAAGCATTTTCAGATTCTTTCATGTGTCAGCTGGACATCTGTAT
GGAGACACCATCAATCAGGACAAAGAGGGAGAACAGTCATTCATCTGTCA
AGGAAACACAGTAGTGCTTCTCTTCAAGAACAAAAGAATAATGTCATCAC
ATCTAATCTCATGCACACACATTTATTTACATCATTTTGCATCCAGGTTA
GGAAGTTGACTCCTAGCACTAGAATGAAAATTGTCTGGAATGACCCCAGT
TGGGCACATATAAGTTTTAGAAGTGGCTTCAATTCATTTTATAGTAAGTA
AGCATTGCTTGTGGAATAAGGCCAAGGTTGAGAAGGCACTGATAATTCAA
GTGCTGCATGGCATTTAGTATGCAGGAATGAAGTAATAAAAAGACCTTTA
CTACTGAAGTACACAGGTAGACAAGGAACACAAAACTTATGTTTGGAATT
TCCTCAGGAAGTAGAATCCCAGGGTGCTGAGCATCATCTATTCTATTCCT
AACAAGGGATATAAGAAAGAGAAGCAAGTGACTTGGTGGTTATGTGACAG
AAGTTAGTCCCCTACCAGGGAAACAGGTTGGTACATTGGGAAGGTGAAAA
TAAGTCAGGAAATTGGGGAAACTCAGTACACAGAAACTTGGTTAGTTCTT
CATTTTCTCACCTCACATTCTTCTTCTCAAAACAATTAAACTCTAATTAA

GCTGGAAATAACTTCTGAGCAAATCAGATGTCGTAACTGTGGGAGAGAGG
GCAGCTGTACTTCAGGGACTGAGGCCCTGTGGCCTCCCTTCCCCCTGACA
CTGTGACAACATGAAACCTGAAGTCTGCAGTACCTGATGGCAGCCAAGAG
TCCCACAGATCCAAACAGTACAAATATGACCTGGCAAGTGACACCTTCCC
>V1ri5
TCCACCTTGGGAGTTTTCCTATGTTCACATTAGCATACTATACAAGAAAA
TAAAGAAATGCTTTAAAAAATGTTTAATTGTTTTTCAAATACCCAGTGAC
CTTAAAGAAATTCTGATAAACAGAATGGAAGAAAATTCAGTAAATTCAGG
ACAGAGACCAGAAAGTCAGCAGTCCATGTGAGACATTCAGCAAGAAAAAT
TGAGATTTTGAAGAAAAAAAGCAACAAGTAAAAGGAGAAATGAAAAAGCT
CAGCCAGGAGGGCAGTTGACTCCTCTGCATAAAAGCAGTGGTCAGAGCAA
AAAGCAGACACAATTTCCAAAAGTCTCCCTGCTGCTCTGCCCTGGACACT
ATACCATAGGACCAGACACTGATTCTGGCCCTTGAACAACCCTTATTCTA
GGCACAGTGAGCNNNNNNNNNNNNNNNNNNNNNNNNNNNNNNNNNNCTAG
ATTTGTTCCTTTTTGTGTGTGCTTTACTTCTATATATTTTGCCTGAATAT
ATGTCTGTGTATCATGTGTGCACTTGATGCCCATGGAGGTCAGAGGCAGA
CTTAAATTCTCTGAAGTTCTATATAGTTGTAAACCACCATATGGGTGCTT
GGAATTGAACCTGGTTCCTCTGCAGGAGCAACGTGTATTTAACCACTGCT
CCATCTCTTCAGCTCTGGTGCTGCTCAATTTTGAAATGTTTAAAAAGGAA
CACCCTCACTTTCTCAGAATTTTCCCGCTATGTTCCTGGCTTTGGTGGGA
GAAAAGAGGAAAAATCACATTCACAAGGAAATTCAAGGTACTGTTCATTT
TCACAGCCCTCCTTGTTACCACAGGGACTGTAATTAGGACCAGAAAGTTT
CTGGCTCAGCAGAAGAAAGGGCACCAGAGAGAGCAGAGAGCTGACATCTA
GAATGAAGATTGCAAAAATAGCATTTTCCCCAGGAGAACCCAAACTCCTG
CTAGTGACATCCTATTAGCAGTGAAAGTTGTGAGCACAGAGCTCAAGGAT
>V1rh12
TTTCTATCTATCTTGACATATATAATGTACAACTTAGTTTCTCTGTACCT
GGATTCCCCTGTTCAATAGAGGGTATAGAGATCCCTGCGTCCTCAAGAGC
TCTCATTTATATTGCAGAAGAAGCATTTTCAGATTCTTTCGTGTGTCAGC
TGGACATCTGTATGGAGACACCATCAATCAGGACAAAGAGGGAGAACAGT
CATTCATCTTTCAAGGAAACCCAGTGGCGCTTCTTTTCAAGAACAAAATA
ATGATGTCATAACATCTAACCTCATGCACACACATTTATTTACATCATTT
TGCATCCAGGTTAGGAAGTTGCCACCTAGGCACTAGAATGAAAATTGTCT
GGAATGACTACACTTGGGCACATATAAGTTTCAGAAGTGGCCTCAATTCA
TTTTAGAGTAAGTAAGCATTGCTTGTGGAATAAGGCCAAGGTTGAGAAGG
CACTGATAATTCAAGTGTTGCATGGCATTTACTGTGCAGGTATTGAGTAA
TAAAAAGACCTTTACTACTGAAGTACACAGGTAGACAAGGAACACAAAAC
TTATGTTTGAAATTTCCTCAGGAAGCAGAACCCCAGGGTGCTGAGCATCA
TCTATTCTAATCCTAACAAGGGATATAAGAAAGAGAAGCAAGTGACGAAG
TTGGTCCCCTACCAGGGAAACAGGTTGGCACATTGGGAAGGTGAAAATAA
GTCAGGAAATTGGGGAAACTCAGTACACAGAAACTTGGTTAGTTCTTCAT
TTTCTCACCTTACATCCTTCCTCCCAAAATAATTAAACTCTAATTAAGCT
GGAAAAAACTTGTGAGCAAAACAGATGTAGTAATTGTTTTAGAGAGGGCA
GCTTTACTTCAGGGACTTAGGGCCTGCAGTGGCCTTTCTTCCCCTTGACA
CTGTGACAACATGAAACCTAAATTATGCAGTACCTAATGACAGCCAAGAG
CCCTACAGATCCAGACAGTACAAATATGACCGGGCAAGTGTCACCTTCCC
>V1rf5
AATCCTCAATCCTACACAAAGAACTACAGGAAAACCAGCAAAACTGGTAT
CAGAAGAGAACTCTTTCACAGGAAAAAGCATAAAAATTGTTTGTTCCATG
CCAAATGCTTAATCCTGAAAACATGCATACTTGTACCATTATATAGACTC
CACAGGGTATATGTAGAAATATATATGCATGTGCAAATACATGTATGCAT
GAAGTAATAATTAATGAAAAGAACACCATGAATTTGAAGGAGATGTGGTA
GTGGTTTTTTGGAGATTTGAAAAAAGGGGAGGGTGNNNNNNNNNNNNNNN
NNNNNNNNNNNNNNNNNNNNNNCATCATGCTTACAGCAAAATTGATCAGA
CACACATATTAGCTCTCTTTAAAAATCTATTTTACTTCATTTAGTAATGC
CAAACAAAGATGATAAATGGTATTTTCTCATAAAATATCAATTGAACATT
TGTTACAGTTATAAAAGCAAATAATAATAAATTATTTTGGACAACTTGAA
TTTATTCAGTAAATGCTATTATGAATTTATAGCAGCTGGTTTATTTTGTG
GTTCACTTTCAAAATATCAAAGAATATATGTCATTGAACTTCCAATCTAT
ATATGATGATGACACCCCCTGAGATGAGGAAGGCATAATGAAATTTGTAT
ACAAAACATCTCAAATAATCAAGATATACACTACACATGCATATCTCAAT

TGTAGACCTCAAACAGTCCACATTATTTATCCAAGTTTAAAGGTGATATT
TGGTGACCTCTGGAGAACTGAGAAAGTAGCAACCCCTCTGAAATCTACAA
TATTCTCTTAAGAACTGACAGCTGGAACTTGTAATTGTACTTTGCTAATA
GTCACTAGGGGAGGAAACTTCAGAGCAACATCTTTACCTGCTCAACCCTA
CATGCAATGTCCATTGCTACTGCATGTGAAATAATAATTGCAGGCCTGAA
GGGAAGGAAGTCACAGAATCATAAATATATATCTGAGACAAAGGGTGCTT
>V1rf4
TGACTGTGAGTATCCACTTCTGAATTTACCAGACACTGGCAGGGCCTCCC
AGGAGACAGTTATATCAGGCTCCTGTCAGCAGGCTTCTGTTGGCAATGCT
TACTATCTGGGTTTGGTGGTTGTATATGGAATGGATCCCCAAGTGGGGCA
GTCTCTGGATGGTCATTGCTTCCGGTTCTGCTCCAAACTTTGTCTCTGTA
ACTCCTTCCATGGATATTTTGTTCCCCTTTCTAAAGAGGATTCTGAGCTT
CTGGGCTNNNNNNNNNNNNNNNNNNNNNNNNNNNNNNNNNNNNNNNNNNN
NNNNNNNNNNNNNNNNNNNNNNNNNNNNNNNNNNNNNNNNNNNNNNNNNN
NNNNNNNNNNNNNNNNNNNNNNNNNNNNNNNNNNNNNNNNNNNNNNNNNN
NNNNNNNNNNNNNNNNNNNNNNNNNNNNNNNNNNNNNNNNNNNNNNNNNN
NNNNNNNNNNNNNNNNNNNNNNNNNNNNNNNNNNNNNNNNNNNNNNNNNN
NNNNNNNNNNNNNNNNNNNNNNNNNNNNNNNNNNNNNNNNNNNNNNNNNN
NNNNNNNNNNNNNNNNNNNNNNNNNNNNNNNNNNNNNNNNNNNNNNNNNN
NNNNNNNNNNNNNNNNNNNNNNNNNNNNNNNNNNNNNNNNNNNNNNNNNN
NNNNNNNNNNNNNNNNNNNNNNNNNNNNNNNNNNNNNNNNNNNNNNNNNN
NNNNNNNNNNNNNNNNNNNNNNNNNNNNNNNNNNNNNNNNNNNNNNNNNN
NNNNNNNNNNGATAACAGTCTCTTTTAACTTTAAAATACTCCTTTAAGAG
CTGATAGGAAGATTTGTAGTTCATTATTGAACTACATCATTGAACTATTA
ACAGGGGAGAAAAGTTCAGAGCTTATTTACTGCCTGGTCAAGTCTACCTA
GAATATCTATTGCTACAACAAGTGAAATAGTAATTGTAAGTCTAAGGGGA
GATGAGGTCACAGAATCACATATCCATATCTCAGACCCCGGTACCTCCCT
>V1rc21
NNNNNNNNNNNNNNNNNNNNNNNNNNNNNNNNNNNNNNNNNNNNNNNNNN
NNNNNNNNNNNNNNNNNNNNNNNNNNNNNNNNNNNNNNNNNNNNNNNNNN
NNNNNNNNNNNNNNNNNNNNNNNNNNNNNNNNNNNNNNNNNNNNNNNNNN
NNNNNNNNNNNNNNNNNNNNNNNNNNNNNNNNNNNNNNNNNNNNNNNNNN
NNNNNNNNNNNNNNNNNNNNNNNNNNNNNNNNNNNNNNNNNNNNNNNNNN
NNNNNNNNNNNNNNNNNNNNNNNNNNNNNNNNNNNNNNNNNNNNNNNNNN
NNNNNNNNNNNNNNNNNNNNNNNNNNNNNNNNNNNNNNNNNNNNNNNNNN
NNNNNNNNNNNNNNNNNNNNNNNNNNNNNNNNNNNNNNNNNNNNNNNNNN
NNNNNNNNNNNNNNACAGCACAAGCCATTGGCACTCTGTTTAGGAATTCC
CCCCCCTGTGCCCATATGTTCGAGACGCTTTTCCACTTTCTCCTCTATAA
GATTCAGTGTCTCTGGTTTTGTCTGTATGCTCTGTAAGAAAAATCATTTA
CCCTCAGTGTGTTTGGAGAAGGTGACATTTTCTTAATTTTCTCAAAGGAG
CTGTTACTTTCTAGTTGAGATCCTAAAGCCACTGTCAAGTATGGCAGACA
CCATGAATGCTTTGGAAGCAGGACGGATATCTTGGGGAATCTAGATTGTT
TTTCTATTTCTTTTAAATGAGACATCATTACTTTTACTTTTTTTTTCCCA
CAAGGACTGGGATCAGTTAATCCACACAGACAGGTGTGGCCTGGAGAGAA
TAGCTCACACACGGGAAACCCAGAGTCAGCTTTTCCCACTCTACTGTTCT
CCTGGAAGTAGGAAAGTTTACTTTTCTATTTGATGATGCCACTTTGTAAC
AACTTCAATGTCAGAACTGCCTTTTCCAGATGACAAGATAACTGTAGGTC
TAGAGGGAGATAAGCTGAGGACAGTTCTTCTGTATAACTACTTCTTAGGA
>V1rc26
CCCACCCATTCCCTCCTCATTGCTCTGGCATTCCCCTACACTGGGGAATC
AAGCCTTCATAGAACCAAGGGCCTCTCCTCCTACTGATGTCAAACAATGC
CATCTTCTGCTACATATGCAGCTGGAGTCATGGGTCCCTCCATGTATACT
CTTTGGTTGGTGGTTTAGTTCCTGGGAGCTCTGGGTGGTCTGGTTGGTTA
ATATTATTGTTCTTCCTATGGCGTTGCTTAGTTCAATGGTTGGCTGCAAG
TATCTGCCACTGTATTTGTCAGACTCTAGCAGAGCCTCTCAGGAGACAAC
TATAACAGGGCTGGTCTCTTTTATGATTTTATGGAAGTTGTTGTGGTTGC
CATTGACTTGGGTTCACTTTCCTTCACCTATGCCCATAATTTGAAGGTTG
GACTTATATCATGGTATCTCACGTACATAAAACTTTCTAGGCTTATACTC
TGTCATTGAGCTACATCCCAGCCACATGTCACTTTCTCCTGATAATGTAG
GTAGAGTACTGTATCACTGTGTGCTATGTAAGAAAGATTACTTTCCTGAA
TGTGTTTGGAGGAGGTGGCATTTTCCTAATTTTAACACAAGAGCTATTAA

TTTCCAAGTGAGACACTGAAGTCATTGTCAAGTAATTATGGAGGAGGATC
ATAAATGTTTTGGAAGCAGGATGGATGCCTTGGGGAATCTAAAATGTTTT
CTACTTCTTTTAAGTGAAATATTATTATTGTTGCTTTGTTGTGTTCACAA
GGACTGGGATTAGTTAATTCTCACAGACAGGTGCAGCCTGGAGAAAACAG
CTCACACAGGAGAAACCTAGATCTCAGCTGTACCTATCCTACAGTTCTCC
TGGAGTTAGGAAAGTTCACTTTTCCATTTGAGGGCACCACATTGTAACAA
CCTCAAGGTCACAACTGACTTCTCCGGAGGACATAATTATTATGGGACTA
CAGGGAAATGAGCAGATGCCATTTCTTCCTTATAACTACTTCATAGAGAC
>SimilarToV1rc33
CTTATTGGTCAAGTGTAAACACACACACAGGTTCTGTATGCTAGGTATAT
TATTTTGCATTGTCATTTACAACTTAAGATGCACTTCTGTAGACTTTTCT
GCCTTCTCTATTAACCTGGAATTAGGAAAGTTCACTTTTCCATTTGATGG
GTTTTCTTTGTCACTTGCAGTTTTTCTCTTAGAACTTTCAATCCAGTTTT
TGTTTTATATTCTTAGATTCTTAGTGTTTTAACTATAAGATACATAAAAA
CTTTTGCTTCTGGTCTTGTCCCTTTTGGTTCTGTGTGCTTCTTATAAATG
TATGGGTGTGGTTTTCCGGCAGTATTCTTCTGTGATCTTGGTGAAGGTAT
TTTCTTTGCCATTGACTTGGCATTTTTCTCTCTAGCTTGTGCTTATAATT
TCAAGAGTTGATTTATTTCATGATGACCTATGACAGAATACTTCTACACA
CTTTACCAAATAAACAACATTTATAGACCAATAGTTTAAGTTCTGGTGAT
AATAGAGTTATATTATCTCCCATCTTTTTGCTTTGTAAGAAAAATGGCTT
TCACCAAGTATGTTTGGTAGAGGTAACATTTTCATAATTTTAGCAAAAGA
GATGCTAAATTTTAGGTGAATCCATGAATCCACTGTCAAGTATGTATAGA
GGATACCATGAATGTTTTAAAAGCAGGATGGATACCTTGTGGAATGTAAA
ATGTTTTTCTACTTTTTAAAAATTGGATATTATTGTTACTTTTGTTTGTT
CACAAGGACTTGTATCAATTAATCCACACACACAGTTAATGCCTACAGAG
AACAGCTTACACCTGGGAAACCCAGAGCTCAGCTGGTTCTACTCCACTGT
TCTCCTAGAATTAGGAAAGTTCACTTTTCAATTTGATAGTGCCACTTTCT
AACATCCTTAAAGTCAAAGCTGCCTTCTCCAGATGACATGATAAGCATTC
GTGTAGAGGGAGATGAGATAATAACCTTTCATCTATATAACTACTTCTTC
>V1rc28
AANNNNNNNNNNNNNNNNNNNNNNNNNNNNNNNNNNNNNNNNNNNNNNNN
NNNNNNNNNNNNNNNNNNNNNNNNNNNNNNNNNNNNNNNNNNNNNNNNNN
NNNNNNNNNNNNNNNNNNNNNNNNNNNNNNNNNNNNNNNNNNNNNNNNNN
NNNNNNNNNNNNNNNNNNNNNNNNNNNNNNNNNNNNNNNNNNNNNNNNNN
NNNNNNNNNNNNNNNNNNNNNNNNNNNNNNNNNNNNNNNNNNNNNNNNNN
NNNNNNNNNNNNNNNNNNNNNNNNNNNNNAATTAGGGACGATTTTCTTCTG
TTATCTTTTTGAAGATCTGGTCCATGCCATTTGACATAGGATTCTTCTCC
CTTACTTGTACCTATAATTTGAAGGGGTTTTTGTTTTTGTTTTTTACTGT
GTCCCACATGCACAATACTGTNNNNNNNNNNNNNNNNNNNNNNNNNNNNN
NNNNNAGTCACTGAATTGAATTTCTGCTGATAATACATTACCTCACATAT
GTGTGCTCTCTAAGAAAGATTACTTTTCCTGAGTGTGTTTGAAGGAGTTA
ATATTTTCTTAACTGTATCAAAAGAGCTGTTATTTCTAGGTGAGACCCCA
AAGCCACTGTCAATTAAACATGGAGACCTCCATGAATGTTTTGAAAGAAG
GAAAAATACTTTGGGGAAGCTAAAATGTTTTGGGGAAGCTAAAATGTTTT
ATTACTTTTTAAAGTAGGGTGTTGTTATTGTTACTTTTGTTCTGCTCATG
AGGACTGGGATCACTTAATCCACACACTGCCTGGAGAGAATATCTCACAC
ATGGGAAACCCAGAGCTCAGCTGTTCCAACTGTACTGTTCTCCTGAAATT
AGAGAAGTTGACTTTTCCATTTGATGGTGCAACGTTGTAACAACCTCAAG
GTCAGAACTGACTTTTACAGATGACATGATAATTATCTGTCTAGAGAGAG
ATGAGCTGATTCCATTTCTTCCTTATAACTGCTTCTCAGGGCCTCATTCT
>V1rc19
GGATGGATGGATGAATGGATGTGACAACGGAGGAGGTATGGGATGTGAAC
AGTCAGAGGGATGATTGGGATAGCCATAAAATCTGAATTTTAATGCAAAA
GAAAGATTAAATAAAGTTAAATAGAAAACTAACAAACAAACAAAAAGGCA
CATGAAAANNNNNNNNNNNNNNNNNNNNNNNNNNNNNNNNNNNNNNNNNN
NNNNNNNNNNNNNNNNNNNNNNNNNNNNNNNNNNNNNNNNNNNNNNNNNN
NNNNNNNNNNNNNGTGGGCTTCTTAAATAATATGGTTGTGTCTTTGGGGCA
GTTTTTTTTCCCCTATGATCTTATTGAAGTTCTTGTGTTTGCCATTGACT
TGGAATTCATCTCTGTCTCCTGTGTATATAATTTGAAGGTTTGATTTGTT
TTATGGTGTCCCACATCCACAACACTTTCTAGGCCTACACTAGGCCAACT
GAACTATATTTTACAGTCCCGCAGCCTGATTTCTTCTGAAAATAACTGTT

ATACTATCTCCTGTGTGTGTGCTCTCTATTAAACATCACTTAACCTTAGT
GTGTTTGAATGAGATGACACTTTTCTAATTTTCTCTAAAGAGCTGTTAAT
TTCTAGCTGTGACACTGCAGTCACTGTCATATACTTATAGAGGGCACCAG
GAATGCTTTGGAAGCAGGATAGATATCTTGGGGGAATATAAATTGATATT
CTACTTCTTTTAAATGGGATATTATTATTCTTACTTTTGTTTTGTTTACA
AGGTTTGGGATCAGTTAATTTACATACACAGATATGGCCAAGAGAGAAAT
CTCACACATGGGAAACCCAGAGCTCAGCTGTTCCTATACTACTGTTCTCC
TGGAATTAGGGAAGTTTACTTTTCCATTTGATGTTTACAGTTTATGACAA
TGTTAAGGCCAGAAGTGCCTTCTCCAGATGAAGTGATAACTATGGGTCTG
GAGGGAGATAACCTGATGACATTTCTCCCATATAACTACTTCTGAGGGCC
>V1rc33
GACTTGAATNNNNNNNNNNNNNNNNNNNNNNNNNNNNNNGCTTCTCTATGC
TAGGTATATTATTTTTCATTGTCATTTACGACTTGAGATGCACTTCTGTA
GACTTTTCTGCCTTCTCTATTAACCTGGAATTAGGAAAGTTCACTTTTCC
ATTTGATGGGTTTCCTTTGTCACTTGCAGTTTTTCTCTTAGAACTTTCAA
TCCAGTTTTTTTTTTTTTTTGTATTCTTAGTGTTTTAATTATAAGATGCA
TAAAAACTTTTGCTTCTGGTCTTGTCCCTTTTAGTTCTGTGTGCTTCTTA
TATTTGTATGGGTGTGGTTTTTCAGCAGTATTCTTCTGTGATCTTGGTGA
AGGTCTTTTCTTTGCCATTAACTTGGGATTTTTCTCTCTAGCTTGTGCTT
AAAATTTCAAGATTTGATTTATTTCATGATGTCCTTTGATAGAATACTTC
TACACACTATACCAAGTAAACATTTATAGACCTATAGCTTAATTTCTGGT
GATAATAGAGTTATATTACCTCCCATATTTTTGCTCTGTAAGAAAAATGG
CTTTCACCAAGTATGTTTGGTAGAGGTACCATTTTCATAATTTTAGCAAA
AGAGATGCTAAATTTTAGGTGAATCCATGAATCCACTGTCAATGTATAAA
GGATACCATGAATGTTTTGAAAGCAGGATGGATCCCTTGTGGAATGTAAA
ATGTTTTTCTACTTTTAAAAAATGGGATATTATTGTTACTTTTGTTTGTT
CACAAGGACTTGTATCAATTAATCCACACACACAGTTAACACCTAGAGAG
AACAGCTTACACCTGAGAAACCCAGAGCTCAGCTGGTTCTACTCCGCTGT
TTTCCTACAATTAGGAAAGTTCACTTTTCAATTTGATAGTGCCACTTTCT
AATATCCTTAAAGTCAAAGCTCCCTTCTCCAGATGACATGATAAGTATTC
GTGTAGAGGGAGATGAGATAATAACCATTCATCTATATAACTACTTCTTC
>V1rc25
CCAAGGGTCTCTCCTCCTATTGATGTCAAACAATGGCATCCTCTGCTACA
CATGCAGCTGGAGTCATGGGTCCCTCCATGTATACTCTTTGGTTGGTGGT
TTAGTTCCTGGGAGCTCTGGGTGATCTGGTTGGTTAATATTATTGTTCTT
CCTATGGGGTTGCAAACACCTTCAGCTCCTTTAGGCATTTCTCTAACTCA
TCCATTGGGGTCTCAGTGCTTAGTTCAATGGTTGGCTGCAAGCATCTGCC
ACTGTATTTGTCAGACTCTAGCAGAGCCTCTCAGGAGACAGCTATAACAG
GGCTGGTTTCTTTTATGATTTTATGGAAGTTCTTGTGGTTGCCATTGACT
TGGCTTCACTTTTCTTCACCTATGCCCATAATTTGAAGGTTGGACTTATA
TCATGGTATCCCACATGCATAAAATTTTCTAGGCTTATACTCTCATTGAG
CTACATCCCAGCCACATGTCACTTTCTCCTGATAATACAGGTAGTGTACT
GTATCACTCTGTGCTATGTAAGAAAGATTACTTTCCTGAGTGTGTTTGGA
GGAGGTGGCATTTTCCTAATTTTAACACAAGAGCTATTAATTTCCAAGTG
AGGCACTGAAGTCACTGTTAAGTAATTATGGAGGGGGATCATAAATGTTT
TGGAAGCAGGAAGGATGCCTTGGGGAATCTAAAATGTTTTCTGCTTCTTT
TAAGTGAAATATTATAATTGTTGCTTTGTTGTGTTCACAAAGACTGAGAT
CAGTTAATCCTCACAGACAGATGCAGCCTGGAGAAAATAGCTCACACAGG
AGAAACCTAGATCTCAGCTGTACCTACCCTACAGTTTTCCCGGTGTTAGG
AAAGTTCACTTTTCCATTTGAGGGCACCACATTGTAACAACCTCAAGGTC
AAAACTGACTTCTCCAGAGGACATAATTATTATGGGACTACAGGGAGATG
AGCTGATGCCATTTCTTCCTTATAAATACTTCATAGAGACTAATTCTACC
>V1rc22
ACAAAATTATAGAANNNNNNNNNNNNNNNNNNNNNNNNNNNNNNNNNNNN
NNNNNNNNNNNNNNNNNCAGAACTCCTGGAGAAATCAGCTGTTCCAACTC
AACCATTTTCTTCTAATTTGGAAAGTTCATTTTTCCACTTAATGGGTTTT
ATTTATTTGTGACTTAACAGAGCTAGCATTCCACTTTTTAATTCTGTATA
TTTAGTGTTTTCTCTATAAGATGCATGGAAATTTTTTATTCTAGTCTTGT
CTTTTTATGTGGTCTCTGTGTTTCTTGTGTTATATAGGTATGTCTTCGGG
TTGGTTTATTGTATGGTCTTATTGAATGCCTTATGTTTGCCATTGACTTG
GGATTATTTTCCATCACCTGTGCCTATAACTTGAAGGTTTGACTTATTTC

ATTGTGTCCTAAATACAGAATACTTTCTAGGCATGTAATCTATGAACTGA
GCTATATCCCAGGCCCACAGCCCAGTTTCTCCTGAAAATACAGTTACATT
TTCTCACTTCTATCTGCTTTGTAAGAAACATTTCTCTCCTAAGCGTGTTT
GGAGGAGGTGACATTTTCTTAATTTTATCAAAAGAGCTGTTAATTTCTAG
GTGAGAACCTAAAGCCATTGTCAATTATGTATGGAACTGACCATAAATGA
TTTAGAAGAACAAAGGATACCATGGAGGAATCTAAAATGTTTTTCTGTTT
CTCTTAAGTGGGTATTAATGTTGTTACTACTGCTTTGTTCACAGGAACTG
GGATCAATTAATCTACACAGACAGATGTGGCCTCCAGAGAATAGCTTACA
TGTGGGAAATTCAAAGCTCAGCTGTTCCTACTCTATTGTTTTCTTGGGAT
TAGGAAAGTTCACTTTTGCATTTGAGCATAGCACTTTCTAACAACCTCAA
GTTCAGAACTGCCTTCTCCAGATGACATAACTATGGGTCTAGAGGGAGAT
GAGCTGATAATATCTCTTCCTTATAACTACTTCTTAGGGCCTCATTCTAC
>SimilarToV1rc16
AAAAACACTAGGACANNNNNNNNNNNNNNNNNNNNNNNNNNNNNNNNNNN
NNNNNNNNNNNNNNNNNNNNNNNNNNNNNNNNNNNNNNNNNNNNNNNNNN
NNNNNNNNNNNNNNNNNNNNNNNNNNNNNNNNNNNNNNNNNNNNNNNNNN
NNNNNNNNNNNNNNNNNNNNNNNNNNNNNNNNNNNNNNNNNNNNNNNNNN
NNNNNNNNNNNNNNNNNNNNNNNNNNNNNNNNNNNNNNNNNNNNNNNNNN
NNNNNNNNNNNNNNNNNNNNNNNNNNNNNNNNNNNNNNNNNNNNATAAGT
ACAGAATATTTGCACACTTCTTTTTTCAAAAAAATTATTTTGCACACTCC
ATGTTCCATTTCCCACGCCCCCACCCCCCATCCACCCTCTGACTGCTCTA
CATTCCAGTGGCAAAGAAGTCTTTGCCAATGACTTCTTTTGCCCTCTGGC
CTGTGACTATAATTTTAAGTTTTGATTTACTTTATGGAGTCCAACCTACA
CAATACTTCTATGCACTCTATCAAATAAACCACATTTCTAGTCCAACAGC
CTGATTTCTGCTGATGACAGAGTTATATTACCTCCTGTCTATTTGCTTTG
TAAGAAAAATGGCTTTCCTCAAGTATGTTTGCTGGAGGTAACATTTTTAT
TATTTTAGCAAAAGAGATTAATTTTTAGGTGAGTCCATGAAGCCACTGTC
AAGCACATATGGAGTATGCCATGAATGTTTTGGAAGCTGGATGTATACCT
TGGAGGATGTAAAATGTTTTTCTACTTCTTTTAAATGGGATATTATTGTT
GTTAGTTTTACTGTTCACAAGGGCTTGTATCAATTAATCCATACATATAG
TTAATGCCTAGAGAGAAAAGCTCACATCTTGGAAACTCAGAGCTCAGCTG
TTTCTATTCCACTGTTCTCCTGTAATTAGGAAAGTTCACTTTTCCACTTG
GTGGTGCCACTTTCTCACAACCATAAAGTCAAAAGTGCCCTCTCCAGGTG
>V1rc12
CAGTTATAATTAGAGCTGAAATAACTTTATGATAAATTATAAAAAAGGAG
ATGATGACAGTTATTTGTCAATAAGAGACATTTGTCAGTTTCTTCTTTAT
CTGAGTATATGATATAAAATATCCTGTTAATACAATCACCAGAGTTATTT
AAAGAATAGGCATGGAAGTATAGTCCATAGCAGGAGCCTATAGGCTTTTG
TCTAAACTGTGGAGGAAGGATGATTTATTTTTTTCCTGGTGGAGAAATGC
AGCATGCTCTGCCAGAAGCATCAAACTGAACATGTAGCAAAGAGCAGTCT
TGAATATCTGATCCTCCCACCACTACTTCCTGACTGTAGTAATTACACAT
GTGTACATTACTATGTGTGATAATACATACAATAGTTACTGTTATGTGGT
GCTGGGTTTCAAACCCAGAGATTCATCCTTTGTACATATAGAGGCACGAA
CTCTACAGACTGACCTACATTCCAGCCCCATAGCCCATTTCTACTGACAA
TACAGTTGCATTCCTTCACCACTGTGTTCTCTGTAAGAAAGATAACTTTT
CTTAAGTGTTTTATGAAGGAGGTGACATATTTGTAATTTTAGCATCTGTG
TTATTAATTTCTAGATGTTACCCTGAGGTATTGAGGCTACAGGGAATGTT
TTACAAGAAAGTGGGGCACCTTGGGAAAATCTGAAATGTCTTTGTACTTT
TGTTAAAGGAAATATTATTGTTACTTTTATTGTGTTCACAAGGACTGGGA
TCAGTTAATCCACACAGACAGATATGGCCTGGAGAGAATAGCTCACACAT
GGGAAAGCCAGAGCTCAGCTGTTCCTACTCTACTGTTCTCCTGGAAGTAG
GGAAGTTCACTTTTCCATTTGATGGTGCAACTTTGTAACAACCTCAAGGT
CAGAACTCCCTTCTCCAGATGACACCATAACTATGGGTCTAGAGGGAGAT
GAGCTGATGACATTCCTGCTTTATAACTACTTCTTAGGGCCGCATTCTGT
>SimilarToV1rc13
GGTGGCAGTCATAATTAGAGCTGAAGTATACTTTATGATAAATTATAAAA
AGGGAGTCATTGACAGTTATGTGTCAACACCAAGAGACATCTTCTTTACC
TGAGTATATGATATAAAGTATCCTGTTAATAAAATCACCAGAGTTGGCCA
AGGAGTATACATGGAAGAACAGTGCATAACAAGAGCCTGGAGGCTTATGT
CTATACTGTGGAGGAGGGAAGTTTTGTATTCCTCCTGATGGGGAAATGCA
GCATGATCTCCCAGAAGCCTCAAACTGGCCATGTAGCAAAGGACAGTCTT

GAATATCTGATCCTCCCACCACTACTTCCTGAGTGTAGTGATTATATATG
TGTACATTACTATGTGTGGTAATACACACAATAGTTACTGTTATATGGTG
CTGTGTTTCAAACCCAGATATTCTTCCTTTGTGCATATAGAGGCACGAAC
TCTATAGACTGACCTACATCCCAGTCCCACAGCCCATTTCTCCTGACAAT
ACAGTTGCATTACTTCACCACTGTGTTCTCTGTATGAAAGATAACTTTTC
CTAAGTGTCTTTGGAGGAGGTGACATTTTTGTAATTTTAGCATCAGTGTT
ATTAATTTCTAGATGAGACCCTGAAATATGGAGGCTACCATGAATGTTTT
GCAAGCAGGAAGATCACCTCGGGGACTCTGAAATATCTTTGTACTTCTGT
TAAGGGCAATGTTTTCCTTGTTACTTTTGTTGTGTTCACAAGGACTGGGA
TTAGTTAATCCACACAGACAGATATGGCCTGGAGAGAATAGCTCACACAT
GGGAAACCCAGAGCTCAGCTGTTCCCACTCTACTTTTCTCTTGGAAGTAG
GGAAGTTCACTTTTCTATTTGATGGTGCAACTTTTTAACAACCGCAAGGT
CAAAACTCCTTTTTCCAGATGACACCATAACTATGGGTCTAGAGGGAGAT
GAGCTGATGCCATTCTTTCTTTATAACTAGTTCTTAGGGCCTCATTCTAT
>V1rb9
ATGCAGAAGCCTCAGCACCCTTCAATGTGTATATTATATTCAGTTCAAAA
AGAAAGTGATGTCATTGTACACGGTTAAACAAGGAAGCAGGTTATTGCAT
ACAGTTGAGCAGGGAGATAGCTTTAGCTCATCTCAGGAGTAAATGTCTCC
GTAGGGGGCACACTTCAGGCTGTAAACATCTGGGAAAGTGTAAGCTATGG
TGGTCATTCTCTGCACACACTGAGAACATTCCTATTTGGACCACAGGAAG
GCCTTGCCATCCACTGAGCTTTACCTAGGGAAGGTTTTGCCCTTACCATG
GGTCTGAGCCACTGATCATTGACAGAGCTGTGCCAATGTCAAATTACACA
ATTTGTTCAAAACTTGGGTGGTTTCCTACAGAATCATCCATGAGTCAGAT
CTCTCTTAACCTCTGAGAAATGTTCCAGTTCATTTGGAGAACAGCGCTGT
CATGGTGGGAAAGCATCAGGCATGGCTGCTGGAATAAGAAACTGAGAAGT
CACATCTTGAACCCAAACAGAAAGCAGAGAGTGAACAGAAGGTGGTAGTA
GCATTTTAGGTTAAACATGGCCTGTGCTGGTTTCAGGTGCCCATGACTGC
CTCACCAAAGACCTCTAGCTCTGCCTCTCTCCCTCTACACTTTCCTATCA
TTTCTTTTTAAATTTAATTGTAAATGTCACTCACAGCTGCAATCCTGAAG
CTGAGCATATAGAAATTAAGACACATAGAACCAATATTACATGGAATTTA
GATACTTCATCTGAGGGTTGACCCTGCAAAAGGTTGCCTGTTAACATTGA
AGGACAAGAGCCTCAGAGCCAATGGGAAGTCATGGGAGGCAGAAGAGTCT
CTCACCCCAGAGCAGACACATCACAAAGTATGACATGTTTTGGGATTGAT
TATACCTGAGTTGTCTAATTGGTGCAAACTTTCTGATCCTTCCCACAGGT
ATTGCAAGATAATTCAGATAAAGTTTGTGCCTAGCCCTCCCCAGATAGTT
>V1rb4
AAGGAGAGTCATCAAGCTTGCTCTGTGAAACATGTCAACATTGAGACCTA
ACCAGGATTTCACTGGCAGTGCATCAGTTTTCTGAGTTCAGCCCCTTCAA
CTTGTCTGTGTTCTGGGTATCAACATAACTTCCCCTATAATCTCCTTGAA
GACTGATCTTTGATAACAGCATCTCTTGATAGAAATTTGGAGTAACAGTC
AAATCATAATTTTGTTATGATCCCTGTCCAGCTTCTGATGCCAAGTTTTC
ATCATTTCATGAAAATCTCAATGTCCTGCTGCAACTGAAAATCTAGTTTC
AGCCTGCAGATTGATTTCATTCTGTTAAGGTCTCTTCTTAATGAGAACTC
ACATATTGAACCCATGCATAGAATAGAGAGTGAACAGGGGGTGATATTAG
GGTTTTAGATTAAGCCTGCACTAAGATGCTTTCATGGGCCCCTGAATTCC
TCACCAAAGGCCTCCAGCTGTGCCTCTCTCCCTCTTCAGTTTCCTATAAT
TTCCTTTTAAATTTACTTGTAAGTTTCACTCAGAGCTTGAATCCTGAAGC
TGAACATATGGAAATTAAGGCTCATAGAACCAATACTACACGGAATCCAG
ATATTCCACATGGGGACTGACCCAGCAGAAGGCTGCCTGTTAAGACTGCA
GGGCGAAAGCCTCAGAGCTGATGGGAAGTCATGGAAGGCCCAGGAGTCTC
TCACCCAAGAGCAGACACATCACAAAGTATGGAATGGTTTGGGATTCATT
ATACCTGAGCTTCAAAATGAGCAAAGCTAGAGTTTAGTTTGTGCGGATTT
TCTGATCCTTCCTACATGTATTACAAGTTAACTCAGACAAAGTTTGTGCC
CAGTCCTCCCCAGGTGGCTGTAATTCAGGTCACAGGGATTAAGATCAAAG
GAAAGGAAGATGCTCCACTGGGATGCCATGTCTCCCTGCTCTCCAGTGAG
AACTTGTCACATTCTACAGAAACTAAAATTACAACTTGCTGAGCTGACAT
>V1ra2
TAATATGAGATAACCCCAATTCTTTTAATTGTGATTCTATGTATACCAGA
CTTCACATCATGAAGGAAGTAGGAGTTAGCTCCTGCAGACGTTTGTCCAT
TTTGTTCTTCTATTGGAGGCAGTTCTATATTGAGATTAGAAACATGGAAG
ATGAAGTTCCAGAGTATCACTCAAACACTGAATTTTCCACACAAGTACTC

TAGAGAGGACTTCACTCGAATATTCTGCTGNNNNNNNNNNNNNNNNNNNN
NCTTCTGTTTCAAACAGGGATGAAGCTGAACAGTAACTATGTATTGCCCT
TTCTCTCCCAGAGCACAAACATAAAGCACTGCATGGGAGAACCAGCAAGT
GAAATATCTGCAGAGACCATCTTGTGCTGAAACACTGACATGTTTGGTGT
GTAAACAGCATCCTCTTATATACCTACTGCCAACCTGACACAGGAACATG
GTTGTGTATATATTCAGGAGGAGGTGACTGAGCTCAGCCTCAGAGTATTC
TGGGTCATACTTGGTTAAAATGTCAGATTGTGGGAGGAAGGAAAAACGTC
TGGATTCACAGATCACAGTTCCCAATATTTGATACATGAATATATTAAGA
AATTTGATTTTCACAAAATATTGAGTAATGATATACACAAATTGAATAAG
AGCATACCCATTCCACAGGAATTATTTTATTGGAAAGAACTGGTTTCTTT
CACACAATCCTCCTCATTAAAACTCACCCAAAATATAGTAAAACTACCTG
TAATGTTCCCGGAAGTGCAGAAGTTGTTGATGAAAATCAAGTAAGATGCT
CCAGGAAAACCTACACATGCTAAATAAGAAAGACATAATCTATGATGCAG
GCAGTATTTAATGTTGAGGGAGCTCACGCACTGATGCAGCCACCATAGAA
CACAGGATCCATCCATGACTCAGCTGAGAACAAGTGAGAGCATGAGTGTG
GTCTGTGGGGTGTAGGGAGGAGCTGAGGTTTTCAGTAAAATGTTGTGAGG
>V1rb8
CCTGAGACCTAACCAGGATTTCACTGGAAATGCATAAATTTTCTGAGCTC
AGCACCTTCTACTCATCACTGTCCTGTGGGCATGAACAAAATATCTCCTG
CAATCTCCAGGAAGACCAATCTTTGACAACAACATCTCTTTAGAGAAATT
TGGAGTTAAACTCAAATCTTGATTTAGTTATGATCCTTCTCCAGCTTCTG
ATGCAAAGTTTTCATTCTTTTGTGATAACCTCAATGTCCTGCTGCAACTG
AAAGTCTAGTTTCAGCTTCCAGATAAATCTCATTCTGTCAAGGTCTCCTC
GTAATGAGAACTTACATCTTGAACCCACACATAGAATAAAAAGTGAACAG
AATGAAGATTGGATGAATAAATATGGGTGGTATTAGTGTGCTAGATTAAA
TGTGCACTAATGTGCTTTCATGGGCCCATGAGTTCCTCACCAAAGGCCTC
CAGCTCTGCCTCTCTCCCTCTACAGTTTCCTATCATTTCTTTTTAAATTT
ACTTGTAAATGTCACTTGCAGCTGGAATCCTGAAGCTGGGCATATGGAAA
TTAAAGTACACAGAACTAATACTTCATGGAATTCAGATATTCCACATGAG
GACTGAGCTAGCCAAATGCTGCCAATTATCACTGTAGGGCATGAGTCTCA
GAGCTCATGGAAAGTCATGGGAGGCCCAGGAGTCTTTCACCCCATAATAG
TTTCACATCACAAAATATGAAATGGTTTAATATTGATTACACCCGAGGTA
CAAAATGAGCAAAGTTAGAGTTGTCTACTTGGTGCAAACTTTCTGCTCTC
TCTCGAGGGTATTGCAAGATAACTCAGATAAAGTTTGTGCCAAGCCTTCC
CCAGATGGCTGTCATTCAGATCACAGGGAGGAAGATCAAAGGTGAGGAAG
ATGCTCCACTGGGATGTCAGGTCTCCCAGCTCTCCAGTGAAAACTTTCAC
CTTCTACAGACAGTAAAATTACAAATAGCTGATGCACATTAAAAAACTCC
>V1rb2
GGGGCACTCTTCAGGATGTAAACAGCTAGAGTGAGGGAAGCTATGGTTGG
GCATTCTTTGCACACCCTGAGAGCATTCCTATTTGGACTAGAGAGAGGCT
TTGCCATGGCACGGAGCTTTATCCAGGGAAGGTTTTGCCATTAACATGGG
AATGAGCCACTGGTCATTGACAGAGCTGTGCACACTCATTCGGGACTTGG
CTGGTTCTCTACAGAAACATTCATGAGTCAGATCTCTCTTAACCTCTTAG
AAATGTTCCAGTTCATGGTTGGAACAGTCTTGGTGGGAAAACATGCCAGC
AAGCAGCAGACATGGCAGCTGGAATAAGAAACTGAGAAGTACATCTTGAA
CCCAAACATAAAGCAGAGAGTGAACAGGAAGTGGTAGTAGGATTTTAGGT
TTAATCTAGTCTATGCTGGTTTCAAGTGCCCATGACTGCCTCACCAAAGG
ATTCCAGCTGTGCCAATCTCCCTCAACACTTTCCTATCTTTTCTCTTTAA
ATTTACTTGTAAATGTCACTCAAAGCTTGAATCTTAAATCTGAACATACG
GTAATTAAGGCACACAGAACTAATACTACATAGAATCCAGATGATCCACT
TGAGGACTGATCCAGCCAAGGCTGCCTGCTAACATTGGAGGTCAGGAGAC
TCAGAGCAGAAGGGAAGTTATGGGAGACCCAGGGGTATCTCACCCCAGAG
CAGACACATCACAAAGTATGAAACCGTTTGGGACTGATTATACCTGAGGT
TCAAAATGAGCAAAGTTAGCGTTATGTAATTGGTGCAACTTTTTGATCCC
TCCCGTGGGTATTGTAAGATAACTCAAATAAAGTTTGTTCCTAATCCTCC
CGGATTGCTGTTATACACATCACATGTGGAAGAGCTTACATTAGAGTAAG
ATCAAAGGCAGGAAAGATGCTCTACTGGGATGCCAGGTCTCCATGCTCTA
CAGAGAGAACTTGTCATGTTTTAAAGACACTAAAATAACAAATTGCTCAC
>V1rb1
GGTCAGTCTTCAGGCTATAAACATCTAGAGTATGGAAAGCTATGGTTGAA
CATTCTTTGCACACACTGAGAGCATTCCTATTTGGACCACCGGAAGGCTT

TGCCATGACACCAAGCTTTATCCAGGGAAGGTTTTTCCATTACCATTGGA
CTGAGCCACTGGTCATTGACAGAGCTGTGCCAAGATCAAATTACACACAC
TCATTTGGGACTTGGCTGGTTCCCTACAGAACCATTCATGAGTCAGATCT
CTCTTAACCTCTAAGAAGTGCTCCAGTTAATTTGGCAAACAACACTGTCA
TGGTGAGAAAGCACCCAACAAGCAGCAGGCATGGCAGCTGGAATAAAAAA
TTGAGAAGTCACATCTTGAACCCAAACATAAAGCGGAGAATGGACAGGAG
GTGGTAGTAGCATTTTAGGTTAAACATGACCACTGCTGGTTTCAAGTTTC
CATGACTGCCTCACCAAAGGCTACCAGCTGTGCCTCTCTCCCTCTACCCT
TTCCTATAATGTCTTTTTAAATTTACTTGTAAATGTCACTCAAAGCTTGA
ATCTTAAATCTGAACACATGGAAATTAAGGCACACAGAAGCAGTACTATA
TGGAATCCTGATTTTCTGAGAACTGACCCAGGAAAGGCTGCCAGCTAACA
CCATAGGATGAAAGCCTCCGTGGTAAGTCATGGGAAGCCCAGAGTCTCTC
ACCCCAGAGTAGACACATCACAAAATATGAAATCATTTAGGACTAATTAT
ACCTGAGATTTAAAATGAGTAAATTTAGCATTATATAATTGGTGCAACTT
TCTGATCCTTCCCACAGGTATTGCAAGATATCTCAGATAAAGTTTCTGCC
CAGTCCTCCCCAGATGGTTGCTATCCACAACACAGGGGGACAGATCAAAG
GTGAGGAAGACGCTCCATTGTGATGCCAGGTCTTCATGCTCCACAGAGAG
AACTTGTCATGTTCTACAAACACTAAAATAACAAATTGCTCACATGCATT
>V1ra11
CAATTTGCTTTAATTCTGTAATAAGAGATAACTCCAATTCTTTTAATTGT
GATTATATTTACACCAGACTTCACATCATGAATGAAGTTAGAGAAATCTC
CTGCAGACATTTGTCCATTTTGTTCTTCTATTTGAGGCAGTTCTAGATTG
AGATTAGAAACATGGAAGATGAAATTACAGAGTATCACTCAAACAATGAT
TTTTCCACAAAAGTACTCTAGAGAGGACTTCACTCCAATATTCTGCTGTT
TTGTAATTTATTTTTTTATCTTATGTATCAAACAGGGATGAAGCTGACCA
GTAACTATGCACTGCCCTTTCTCTCCCGGAGCACAAACATAAGGCACTGC
ATGGGAGAACCAGCAAGTGAAATATCTGCAGAGACCATCTTGTGCTGAAA
CACTGCCATGTTTGGTGCTCTTATATACCTAGTGCCCAACTGACACAGGC
ACATGGCTGTGTATATGTTCAGGAGGAGGTGATTGGAGCTCAGCCTCAGA
GTATTCTGGGTCATACTTGGTTAAAATGTCAAATTGTGGGAGGAAGAAAA
AACGTCTGGATTCACAGATCACAGTTCCTAACATTTGATACATGAATATA
TTAAGAAATTTGATTTTCACAAAATATTGAGTAGTGATAGACACAAATTG
AATAAGAGCATACCCATTCCACAGGAATTATTTTATTGAAAAGAACTGAT
TTCTTTCACACAATCCTCATTCAAACTCACCCAAAATAGTAAAACTACCT
GGAAGTGCAGATGTTGTTGATGAAAAGTAAGTAAAATGCTGTTCCTGCTC
CCAGAAAACCTACACATGCTAAATAAGAAATGACATAATTTATGATGCAA
GCAGTATTTAATGTTGAGGGAGCTCACGAACTGATGCAGCCACCATAGAA
CACAGGATCCATCCATGGCTCAGCTGAGGACAAGTGAGAGCATCAGTGTG
GTCTGTGGGGTGTAGAGAGGAGCTGAGGTTTTCAGTAAAATGTTGTGAGG
>V1rc32
GTCATTAAGGACTTGGTATGTATTGCTCCAGACATTTCTGGCTTTGAAAT
TTTATCTCGAATAATCAGCTGTTCCTATTTAAATATTTTACTAGAATTAG
GGAAATTCACCTTTCTATTGATGAGTTTCATTTATTTGCCACTTGCATTT
TCCCTTAGAGCTTTTGACTCACTTTTTTTGGTTTGTTTTTCATTTTGTTC
ACTTAGNNNNNNNNNNNNNNNNNNNNNNNNNNNNNNNNNNNNNNGCATGGATAN
NNNNNNNNNNNNNNNNNNNNNNNNNNNNNNNNNNNNNNNNNNNNNGGTCTT
CTTAAATTGTATGCTTGTGACTTCTATGATCCTATTGAATTTCTTATATT
TGCCATTGACTTGTAATTCTTGTCTGTCACATGTGCTTATAATTTGAAGA
TTTGATTTTTTTAATTGTGTCCCTTATACAAAATACTTTCTGGGCATACA
GTCTGTCAACTGAGCTACTTCCATCTCCACAACCTTATTTCTACTGAAAA
AACAGTCATATTACCTCCTGTGTGTGTGCTCTGTAAGAAACATCCTTTAC
CCTTCATCAAGGATTGACATTTTCCTAATTTTAGCAGATGAGATGTTAAT
TTCTATATGAGATGCTGAAGTCACTGTCAGGTAAGTATAGAGGGCACAAC
AAATGCTTTGGAAGTGCTATGGATACCTTGAGGAAATTAAATTGTTTTTA
TATTTCTTTTAAATAGGATATTATCATTCTTACTTTGTTTTGTTCACAAG
AACTGGGATCAGTTAATTCACACAGATATATGGCCTGAAGAGAATAGTTT
ATCCATGAGAATCCCAGAGCTCAGCTGTTGCTACCCTACTGTCCTCCTGG
AATTAGAGAAATTCACTTTTCCACTTGATGGTACCATTTTATAACAACCT
TAAGATCAGAAGTACCTTCTCCAGATGACATAACTGCCTCTAGAGAGAGA
TGAGCTGATGAAATTTTTTTCCTTATAACTACTTCTTATGGTTCCATTGT
>V1rc3

CTAAGGACTTTATACGTATTGCTGCAGCATCTTCTGGCTTTGAAAGTTTA
CTGTGAGAATTCAGATATTCCAACTGCACTGTTTTCCTGGAATTCGGGGA
GTTGACTTTTCCATTTGATGGGTTTTGTTTATGTGTAACTTGTAGTTTCT
CTCTTAAATTTTTCAATCTACTTTTTTGTTTTGTATAAGTAGTGTTTCTA
CTTAAGATATATTGAAATTTTTGTTTCTCCTCTTGTCTTTTTTTTTGTGTT
CTGTGTGCTTCTTCTACTTGTGTCTTTTCTTACTTTGGAGCACTTTTGTT
CTATGACCCGCTTGAAGATCTTGTGTTTGCTATGTACTTGGGATTTTTTC
CCCTCATCTGTACCTATAATTTGAAGGTTTGATTTTTTTTCATGTTATCC
CACATACACAATACTTTCTACCCATACACTCTGGCAACAAATCTATATCC
CCAGCCCCATAACCCAATTTCTTGTAATAACACATTTACGTTACCTCACC
TCTGTGTGCACTCTAAGAAAGATTGCTTTCCTCAAATGTGTTTGAATGGG
CAGCCATTTTCTTCATTTTAGCAAAAATGCTGTTAATTTATGGGTGAGAC
CTTCAATCCACTGTGAAGTATGTAAGGAGGCTACCACAACACAAATGTTT
TGGAATCAGAACAGATACCCTAAGGAATCAAAAAGGTTTTGCTACTTCTT
TTAAGTGTGATACTATTATTGCTATTTTTGTTTTGTTCACAAAGACTGGG
GTCACTTAATCCACCCAGACAGTTATAGCCTGGAGAGACTATCTCACACA
CGGAAACCCAGAGCTCACCTGTCACTAAGCCATTGTTCCCCTCAAATTAG
AGAAGTTTACTTTTCCATTTGATGGAATCACTTTGTGACAACCACAAGGT
CAGAACTGACTTCTCTGGATGCCATGATAACTACTGGTCTAAAGGGAGAA
GAGCTGATGACACTTCTTCCTTATAACTGCTTCTTAGGGCCTCATTCTAC
>V1rc5
TAAGATTGGTGNNNNNNNNNNNNNNNNNNNNNNNNNNNNNNNNNNNNNNN
NNNNNNNNNNNNNNNNNNNNNNNNNNNNNNTTCCTACTCTACCATTTCCT
GTTATCAGGGGAATTCATTTTTTCATTTAATGGGTTTTATTTATTTGTCA
TTTGAAGTTTTTCTCTTAGAGCTTTCAATCCATTTTTTAGTTTTGAATAT
TTAGTGTTTTACTTATAAGATTCATGATTTTTTTTTTGATTCTGCTCTTT
TCTGTGCATTCTGTGTGCTTCTTGAGTTTTATGGGTGTGTCTTTGGGTTG
CTTTCTTCTATGACCTTATTGAATGTCTTGTATTTGCAATTGACTTGGGA
TTATTTTCCCTCACCTATGCCTTAAACTTGAAGGTTTGACATGTTTCATG
GTGTCTTACATTTATAATACTTTCTAGGTATGGTCTCTTATCAACTGAGC
TACATCCCGATAAACAGTCCTGTTTCTCCTGATAAAAGAATTTCATTACC
TCACTTCTGTGTGCTGTAAAGAACGATTACTTTTCCTAAGTGTATTTGGA
GGAGGTGACTTCTTCCTAATTTTATTAAAAGTGCTGTTAATTTCTAGATG
AAACCCTGAAACCTCTGTCAAGTAAGTACGGAGGGGACCCATGGAGGTTT
GGGAAGCAGGAAGGACACCATGAGAAATCTAAAATGTTTTCCTACTTCTT
TTAATGGGGGCATCATTATTATTGTTATGCTTGTTTTGTATAGGACTAGG
ATCGGTTGATCCACATAGACACATATGGCCCAGAGAGAATAGCTGACACA
TGGGAAACCCAGAGTTCAGCTGTTCCTAATTCACTGTTCTCCTGGAATTA
GGAAAGTTTACTTTTCTCTTTGAAGGTACCACTTTGTGACAATGTCAAGG
TCAGAATTTCCTTCTCCAGATGACATGCTAATTAAGGGTCTATAGAGAGA
TGAGCTGATGACATTATTTCCTTATAACTAGTTTTGGGGCCTCATGCTAC
>V1re9
TAGAGAAAGGACTAAAGGAGCTGAAGGGGTTTGTAACCTCATAGGAAGAA
AAACAATATCAACCAACCAGACTCCCCAGAACTCCCAGTGACTAAACCAC
CAAGCAAATACTATACATGGAGGGATCTGTGGGTCCAGATGGATATGTAG
CAGAGGATAGCCTTATCGGGCATCAAAGGGAAGAGAGGCCCTGGTTCCTG
TGAAGGCTCCATGCCACAGTGTAGGGGGATGCCAGGATGGGGAGGTGGGA
GTGAATGGGTAGATGGGGGAACACCCTTGTAGAAGCGGGAGGGGGAATGG
GATAAGGGGTTTCCATGGGGGGGGTGAATCTGGAAAGGGGATAACATGTG
AAATATAAATAAAGAAAATATCCAATTACAAAAACCAATTTCTTTAAGAA
TTCATAAGTTCAGAGGATAACTATATGTGGAGATGCTTGTGTAGTATGCA
AATACTATTATGCTAAGTAAACTAGGTGGCATTTTTATTTTAAGAACCAT
GAAAATTGGAGAGAAGAGTTGTAAGGGGATGAAAAAGGACTTGATAAGTG
AACAATGCAGAGTGGACTTAGCTAAAATACATATGCATTTATATAATACA
CATTTAAGTAATAAATATTCTATATGTCTTTGAAAAGAACAAAATTATAT
TTTGTCTGAAGAAACCTGAAGGCAATGAAAGAAAGAAAGAGAGACTTCTG
GAAATTTCTCATGAATCTCCCCTCAAAATTTGTAGCACCTTCCATATGAT
ACCTGGTCTATCTTCAGGGATCATTAAAAACTTAACCCCTCTTCAAAACA
TATTGTCCCTTTGGAGATATTCACCAGAAACTCTTAAATGTCTTCTAAAA
TTAGAAACTGGAAAATGTCTCATATGTGATTTTGTCTCCCAAACCCAGCC
CTGCAATTACACTGCAATCAAAATGAAAATTGTAGTTCTAGAGGGAGGTG

AGGTTACATAGTCACAAACCTATATATCAGGCTTGAGCTGACTCTCTTAC
>V1rg1
TACAAGGCTGGACTTATGCCCTGCACAAATGTAGCAGAGGTGTAGTTTGT
TCTTCATGTGGGTTCCACAAAAACTTGAGCAGAGACATACTCTCACTCTG
CTACCTTCCTGTGGATCCTGTTCTCCTAACTGAGCTAGGAGAGAAGTTGT
TTGACCTCAGTGGAAGAGTATAGACCTAGCTCTGCAGTGAAATGTGTTGC
CAGGGTGGGTTGATATCCATAGAGGACACCCCACTTCTCATACATAAATG
AGAGTGGGGATAGGTGTAGGGGCCATGTACAGGGGTAGGGAAGGAATTGG
ATGTAAGGGCTGGCTTTGCTTGGAATTTAACGTGAATATGTAAATTAATT
AATTAATGGAAAAAATAGAGAATAATCAAGAGAAGGTCCACCGACAAAGA
AATAATCCACCGACACAGAAGCACATGGTGAAAATGTCAAGGTTTATAAA
AAAGAGAAGCTGGGCAAAAGGAAATGAATTTCAGGCTCAATAGAGGTTTA
GGGTAGGGGCCTGAGGTGAGAATGCTGATAGTAGCCAGGACTCTGTAGGA
GCTACTTGCAATGCTCTGAGAGTCTGGCAGCTAGCATCGCTTTCATATAT
TAATAGGCACCTAAATTAGCCATTTGTCCTGAGTGTCTTTGGGAGGATAA
AATTTTGAGATGTAGGTGATAGGGAATGACCCTCCAGGAACAAGGCCACG
CAACTTGCTTATCCTTCTCCTTGCCTTTTTGACATACCCTTGGTGCTCCA
AATTCTGACAAAAGTGTTCCAATTCAAATTCCTAATTGCTTCCTTGGAGT
AGTGCGTGTAATTTGCACATCTTGTTTTGTTTGCTTTTGTATTGTTTTCT
GACAGGAAGGTATTTAAAAGTTACTGCAGATCCTTGTTGAGAGGCTGTCA
TCATGGTTCCAGAGTTGAGTTGAAAATGACCAATCTTCTGGGATACAATA
ATAGGAGATCAGGAATCTTTAAGTCAGCACAAGTGTTTCATCAGACTTTC
>V1rg2
TTTTCTGACAAAATACAATTTAGACATGAGCATTCTTCAAATTAAGAGGC
TTCCTAAATGCTACATTTAATACTTGATCATATCTCTTTTTCCCTTTCTT
TTTCATTTTTGAATCATTTTCTTTCTATTTTTGTTAAATTATTTTATTTA
CTTACATTCCAAAAGTTACCCCATTTCCTGGTTGCCATTCCCCTAGATTT
CACCTCCTCCCTCTTGCCCCCTGCTTCTGAGAGGGCATTACCCCATGTAC
CCACCCATCTGTATCCTACCCCCACCATCATCCTTCTTTCCTGGGACATC
AACTTTACACATGACTAAATGCAACCTCTTCCCCTGATGCTATACAAGGC
AGTCCTCTGCTACATTTGTGTGCCCAGGGCCACAAATTAGGTCATATATG
CTCCTTGGTTTTTTACTTAGACTCTGGAAGCTCATCTACAAGGATTAATG
TACAAGGCTGGGGTGAGAAGTGTTGAGAGGAATCAAAACTCTGTAGAGGT
ACTTACAAGGCTCTGAGAGTGTGTCCAGCTAGTATCTCCTTTTAGTGTGT
TAATAGGCACCTAATTTAGCAATTTGTCCTGTGTGTCTATGTAACCNNNN
NNNNNNNNNNNNNNNNNNNNGTGGAAACTATCTGAGAGGCTGGGTGATAG
GAGAAGCCCCTCTAGGGAAAAGTCCATGAAACTTTCTTATACTTTAATTG
CCTTGAAGTTCAGTCTGTGTTTCACTTTGCCGTAAGTTTTCTGCTGCAAG
GACTGACAAAATAGTTCCTGTTCAGATTCCTAATTGCTTCCTTGTGATAG
CTCATGTAATTTGTATATCTTGGTTTTTTAAGTTATGTATTTTTTTCCTG
AGAGGAAAGTGATTAAAAGTTACTGAAGATTCATGTGGAGACGCTGTCAT
CAGGGGTCCAGAACTGAGCTAAAAATCACCAATCCTCTGGGAAAGAAACC
CAGGAGAACTTTACCCCTTTAAGTGACGGGGAGTATTGCACCGTGGTTTC
>V1rg16
AAGGAAAGGAAAGGAAAGGAAAGNNNNNNNNNNNNNNNNNNNNNNNNNNN
NNNNNNNNNNNNNNNNNNNNNNNNNNNNNNNNNNNNNNNNNNNNNNNNNN
NNNNNNNNNNNNNNNNNNNNNNNNNNNNNNNNNNNNNNNNNNNGAGACCA
GGGTGTCTCCTCTCATTGATGACCTACTAGGCCATCCTCAGCTCCACATG
CAGCTAGAGACATGAGTCTCTCCGTGTGGTTTCTTTGATTAGTGGTTTAG
TCCCAGGGAGCTCTGGAGTTACTTTCCTCCTAATGGGCTGCAAACCCCTT
CAGCTCCTTGTGTACTTTCTCTACCTCCTTCATTGGGGACCCTCTGCTCC
ATCTAAAGGATGACTTCAAGCATCCACTTCTGTATTAGTCAGGCACTGGC
AGAGTCTCTCAGGAGACAGCTATATCAGGCTCCTGTCAGCAAGCTCTTGT
TGGCATTTGCAATAGTGTCTAGGTTTGGTAGTTGTTTATGGGATGGATCC
CCAGGTGGTGCTGTCTCTGGATGGTCATTCCTTCAGTCTCTGCTCCAAAC
TTTGTCTCTGTAACTCCTTCCATGGGTATTTTGTTCCCCCTTCTAAGAAG
GATCAAAGTATCTACACTTTGGGTACCAAACACTCTTGTGATGTTAAAAC
TGTCTAAGAGGTTGGGTAAAAGGGAAAGGCCCTGCTTATGTTTATGAACA
ATGAAAAACTATTTCTTCTGCATGTCATTCTGCAGTGCACTTTGTATTCC
AAGGACTTACAGGAGAGTTCCAATCCTGATCCCTAGTTACTTCTTTGAAT
TGCTCTTGTAATTTTCACATCTACATTTTGTTTTGTTTTCTGACAGGAAC

TCAGTACTAATGAGTTTAAAAGTAACTTCAGACCCTTGTGGAAATGCTAT
CATCACATTCAAGTGCTGGGAATAAAATGGCCAATCTTCTGGGAAAGAAT
ACCAGGAGATCATCAACCTTTAAATGAGGAAGTGAGTCTCACCACAGTTT
>V1rg10
CATTTTTGAATGAAAATGTGGATATGACCACACCAAGTTCTCATTGGCAA
GAAGGTTTTTATTGTAGATATGAAACAGAGAACATTCAGAAGCATTTGAA
AGCATCCAAGGCTCTGAGAAATTGTAGCACATTAGACAAGGCCCATGCAC
AGAACCAGGCATAAGAGGAGAGAGGTGAGGAGGGCAGCAGAAGTCAAAGA
AAACACTGCAAAAGAAACTAAAATAGTGCATGAGAAGAATCAAGAAGACA
AGGAACACCCTGAGAAGAAACAAGACCAACAGTTGGTCCTCTCTGCTCAG
CTCCTTTCACTGTCAGGGATGGCTCTATTTTCTTCTAAGCAGTGAGAACA
CGGTTACCCTGTGTCTGGGTAGATATGGGGTAACTGTAGCCCATAAACCA
ACATATCTTGGTTTTAAAGGATAGAGGAGCTAGGGGAAGTGAAGGGAAGA
TGAGAGTATAAAGAGTTTTAATTTGAATGGCAGGGGTTGTGCTGAAAGGA
ACCAAACCCTGTAAGAGGTTGTTTGCAATGCTGTGAGAATCTGGCAGACA
GAATCTGCTGATATGTTAATAGGCCCCTCAGTTAGCCATTTGTCCTGAGT
ATCTTTGGGACTAAACACACTTGAGATGTTGAAACTGTCTAAGATTCTGG
ATGACAGGGTAGGACCCTCCAGAGTCAAGGTCATGAAATTATACTTTCAT
AGCAATGAAACCATGTGCCTTCTGCATGCCTCTATGTCATCCTGTGTTTC
AAAGGCTGAGAGAAAAGTTTCTATTCTAATTCCCAGTTGCTTCCTTGGAG
TTGTTCCTGTGATCCATACTACTATTTGTTTTGTTTTCTGACAGGAACTC
AGGACCAAGGTCTTTAAAAGTTACCTCAGGCTCTTGTGGAGGTGCTGTCA
ACATGGTTCTAGATCTGAGTTTAAAATGACCTGTCCTCTGGGAAAGAATA
TCAGGAGATTTTGAACCTTTAATTGAGGGATAGTGTCTCACCACAGCTTC
>V1rj3
CAGCATTAGCTGGACTTCGACTTGCTATTTAGAGCAGGCTGGCTTTGAAC
TTAAAGAGCTCTCAGCCTCTGCCCCTGCCCCTGCCCCTACCTCCGAAGTA
CTGGGAATAAAGGGACCCTCTACACACAGTCACCCCTCCTTTTTTCTGTG
TTTTTTTGTATTCAGGTCTTGAGTTGTTGATCACCACTATGGGATGATCG
TGGTTGTCATATCCAGGAGACAGCATTTCATAACATTGTTCCTTTGGACC
TTAGAATTTTTGCTCTCTTCTCCCAGTGTCCCTGGGTCTTGAAGACGATA
TAGTAGATGTCATGTTTAGGTATCCAGAATTAATGGACAGTTATTCCAAG
CACTTTGAATACTTGTGAATCTCTCTGTGTTAAATATTGACCTCTGATGA
GAAGTTTTTGTGATCTGGGCAGAACTGAGACTTGTCTGTGGGTATAAGCA
TAAATATTTGGAAGGCAGATTGACTAGTGTGTGTTTTGCAAAACTGAGCA
CTCTTAGAGTCAAACTTTATTGGTAGGCAAGGATATAATACAACGATGAC
TAATCCATGCACTGTGTGGTCAAAATAAAACTCCAAGTGTACCTTTGTAT
TCTCCATGGAACCTTAAAACACAGTGTAAGAACGCAAGGCACAGTGGTCT
CCAGTGGAACTGTTTGAGTGTTTTTTGAGGCTCTGCTTGGAGTTCAGCTT
TTATGCTAACAGCTGTGATAATTCCCACCACTGTTTTCAAACTTTTAATG
ACTTCTTCACTCCTCAAGTTTACTTTCTGATATGTATCATCTTGGGGAAA
TGCAAATATTCAGAAGTAGATTTTGCACTCTTAGAATGCTAAAGTTATCT
GGGTGAGCAATGTTTTCCCCTGAGCATATCTAAGTAATGCGCAGTAACAA
GCTCATTGATGCCTTGGGTGCCAAGCTGGTCCATGTCCACTGGGAGGAAG
AGTCCTGTCATCCTGTCCTTGATTTAGCCATGCCACCTGCTAGGCAGCCT
>V1rk1
GGGGAAGCACCCTCATAGAAGCAAGAGAGGAGGAATTAGATAGGGGTATT
CTGGAGGGGAAACCAGGAAAGGGGTTAACATTTGTTAAAATGTTAAATAA
ATAAAATATCCAATATAAGAAAATATAAGTTCTTACCACCAAAAAAGGGA
TATTTTTAGAGAGAGAGAGCTTAACTTACATCCACTTAGAATAAAAGTCT
CTGAATTCTCAATTGTATATTCTAATCTATACATTTATTTGTTTGCTTGA
TTGCTTGCTAGAGCATCAGGCTATGTAACCCTGCCTGTCAGGAAACTCAC
TGTGAAGACCAGGCTGGCATAGAACTCATGAACATACAGTCATCTCAGCC
TTCCAAGTGTGCCTCAATGCCTGGCAAGTTTTGCTTTTAATTCCAAATTT
GACTCATTAAATCTGAAAGTAAAGGCTATATGAAATGTTCACAGTGGTTG
CTACATGAAGAAAGAAAGGGTTACATTGACATAAGTACACATTAGTGTAC
ACAAACTTACTTGCCATGTGGGGTATGTATAATTTCATGTTGCAGCAAGT
GAGTATCAGAAATACTTAACACAAAAATAGATAAGTACCTTATTATCTTA
GGAAGGAATTTCAAATAAGAGTCTAGGTGAAACAAAGTGTCTTATAGCAA
TGACTAATCCCTGTATGGTGGGCAAAAGTAAAATTCACCATCTGGCATCA
TAGCCTCTGTGGAGTACAGAAAAGTTCAAAGCACACTGTGGGAGTAGGAG

ACATGATGGTTTCCCATGGAACAGAGAGTGTGCTCTCTGGAGTCCTGTTC
ACTGCTCAGCCTGCTTGAATGGCTGTGATGACATCCACCACTACAGAAGA
ACTAACTTTTAATGACTTTTCAGTTCCCAGAGTGGACTCTCTGGTGTAGC
TCAACTTGGGGAAAGGTACGTCCTCAAAGGCACCTGGCTTAGTCTGATTA
ATAAGTTCACCAGGAGACTCATTTTAAAGGTGGAAGACTGATATATTATT
>V1rd13
CTTTAATAAAAATATTGTGCAATAATAGTTTCTCACACATCCAAGTATAT
GTTCTCTGAATTGCCAAAGACAATCTTGCTGCAGGTTTGAATATCAATAA
AGGATTAGAACATACAATTCTTACATTTATAACCTGAGTTTTTACTTTTC
ATCATTGAAAAGCAAAACTAAGTCCTTAGTTTTCTATTATACCTAGAGCA
GAAATAAGAGTGATTGGATATATTTATTTCTTAATGATTGCTCAATCCAA
ATCTTTGCTTGTATGTTCAGAATTTCTTCCTTACAAAGCAGATTCTCTTG
ATACTAGCAATGTATATTAGGCCTCTGATAAGGATTCATGAGTATTTCAG
AAAACAAGTCACAATCTACCCGAAAACAAGTGGATATGGGGATAACTCCA
GGATTCAGATGGGAAAGATCAGCTTCGGTGAATAATTTTGTCAGAAGTCT
GTGTCCTGGGACAAACATGGGAAAGTTGGAATGAATCAACTTCCTGGGCA
ATTACAGAATCGGAATGACATGTTGCCTCTTTTCTGGAAGGAGTTTGTGT
CAGCATAATTTTGCAAGAGTAAAATACTAGGGAGGAACATAATCCATAGA
AATGTTCATAATGAGTCCCTTGTTCTACTCTTGAGTGCCCCCAATTCACA
TCTGATTACAAGATCAATTCAGAACTTAGATTTCCCCAGAGGAAACATGA
AAACTCTTTCATAACTTCTGCTCAGGTACTCATTAAAATTTCACAATCTC
CTAGACATTATCATTAAGCATCAGAAAATAAAAATGGGGATTTTTCTCTG
GGGATAAATGATTTTTCCTTTATAGGAAGTATCCGAAAGACATTATGCAG
ACAAATGCACTGACATCACAAATAACACAATAATAAACTCTGGAAGATGT
CATGTCCAGGCACCCTATTATTTTATGAGAGTGGAAACCTGAACTCTTTT
GGGTTACCTTCACAGAACTGCATGGAGAATAAGAAGCCATTCATAATTTT
