# Supplementary material for: Common Promoter Elements in Odorant and Vomeronasal Receptor Genes
Source: PLoS One. 2011 Dec 28;6(12):e29065. doi: 10.1371/journal.pone.0029065 (PMC3247230; doi:10.1371/journal.pone.0029065)
Supplement: Figure S3 — Motifs consensus sequences. The consensus sequences of the V1R motifs that were used in the bioinformatics analysis are shown. (PDF) [file pone.0029065.s003.pdf]

MOTIF consensus sequences (the sequences follow the IUPAC degeneracy code, shown bellow).

Symbol	Meaning	Nucleic Acid
M	A or C	
R	A or G	
W	A or T	
S	C or G	
Y	C or T	
K	G or T	
V	A or C or G	
H	A or C or T	
D	A or G or T	
B	C or G or T	
X	G or A or T or C	
N	G or A or T or C	

Degenerate motifs used in Siteseer (http://www.chick.manchester.ac.uk/SiteSeer/)

>MV3F
CNTCTGSRRAW
>MV3R
WTYYSCAGANG
>MV7F
TYYMCARRKG
>MV7R
CMYYTGKRRA
>MV12F
YCCAGARSWS
>MV12R
SWSYTCTGGR
>MV15F
CYYNTCTCYCYY
>MV15R
RRGRGAGANRRG

Degenerate motifs used in STAMP (http://www.benoslab.pitt.edu/stamp/)

>MV3
TCNTCTGGRNAW
>MV3R
WTNYCCAGANGA
>MV7
TYYCCAGAKG

>MV7R
CMTCTGGRRA
>MV12
YCCAGARNWC
>MV12R
GWNYTCTGGR
>MV15
CYNNTCTCYCY
>MV15R
RGRGAGANNRG
